# Aging Impairs Macrophage Phagocytosis Through Mitochondrial ROS‐Induced Collagen Production

**DOI:** 10.1111/acel.70594

**Published:** 2026-06-15

**Authors:** Yuming Wang, Xin Xu, Nuanqin Shen, Ping Han, Liuyi Wu, Yi Jin, Yiming Sun, Lanlan Xiao, Jinyou Li, Lan Wang, Yunmei Yang, Qin Zhang, Weiqian Chen, Chaohui Yu, Bowen Wu

**Affiliations:** ^1^ Department of Geriatrics The First Affiliated Hospital, Zhejiang University School of Medicine Hangzhou China; ^2^ Zhejiang Key Laboratory for Diagnosis and Treatment of Physic‐Chemical and Aging‐Related Injuries The First Affiliated Hospital, Zhejiang University School of Medicine Hangzhou China; ^3^ Department of Gastroenterology The First Affiliated Hospital, Zhejiang University School of Medicine Hangzhou China; ^4^ Institute of Immunology Zhejiang University School of Medicine Hangzhou China; ^5^ School of Cellular and Molecular Medicine University of Bristol Bristol UK; ^6^ Department of Rheumatology The First Affiliated Hospital, Zhejiang University School of Medicine Hangzhou China

## Abstract

Macrophages are pivotal immune cells due to their phagocytic capabilities, yet the impact of aging on macrophage phagocytosis remains poorly understood. Using comprehensive in vitro and in vivo phagocytic assays, we demonstrate significantly reduced phagocytic activity in monocyte‐derived macrophages from aged humans and mice compared to young counterparts. RNA‐seq analysis revealed upregulated expression of extracellular matrix protein genes, particularly collagens, in aged macrophages; manipulation of COL1A1 expression can significantly affect phagocytosis. Protein interaction assay identified binding between collagen and actin filaments, which inhibits F‐actin turnover and consequently impairs phagocytic function. Also, we found that mitochondrial ROS is the driving force of collagen overproduction and MitoTEMPO rejuvenates macrophage phagocytosis via restoring actin dynamics. In a mouse model, MitoTEMPO significantly boosted the phagocytosis of peritoneal macrophages against bacteria. These findings highlight the fundamental role of mitochondrial redox balance and collagen production in controlling macrophage phagocytic function, identifying them as targetable mechanisms for promoting healthy immune aging.

## Graphical abstract

Aging compromises macrophage phagocytosis via the mitochondrial ROS‐driven overproduction of intracellular collagen. This excess collagen physically binds F‐actin, paralyzing cytoskeletal turnover; however, mtROS scavenging successfully rescues actin dynamics and restores phagocytic capacity.

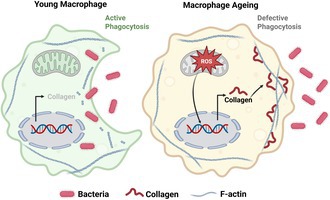

## 1 Introduction

Disease resulting from infection constitutes one third of mortality in the global population aged 65 or older (Kline and Bowdish 2016). Age‐related susceptibility to infectious diseases is characterized by increased pathogen diversity in the elderly (Gavazzi and Krause 2002), reflecting a decline in the immune system's capacity to eliminate microbes at the primary defense barrier. Monocyte‐derived macrophages (MDMs) are pivotal effectors of antimicrobial immunity; they rapidly infiltrate infected tissues to orchestrate early responses and can differentiate into various subtypes to reestablish tissue homeostasis. In aging, monocytes exhibit heightened sensitivity to inflammatory stimuli, adopting a pro‐inflammatory (M1‐like) phenotype with exaggerated cytokine secretion (Hearps et al. 2012). Paradoxically, this hyperinflammatory state coincides with defective pathogen clearance, indicating a fundamental disconnect between inflammatory signaling and other effector functions such as phagocytosis.

Age‐related changes in phagocytosis of macrophages have been reported in several contexts in the literature. For tissue resident macrophages, brain microglia exhibit reduced uptake of myelin (Natrajan et al. 2015; Rawji et al. 2020) and lipid debris (Rawji et al. 2018) with age. Also, aging impairs alveolar macrophage phagocytosis of bacteria (Li et al. 2017) and latex particles (Higashimoto et al. 1993). However, purified pulmonary macrophages from old mice showed enhanced uptake of *M. tuberculosis* and subsequent lysosomal fusion—a phenomenon potentially attributable to increased pre‐existing monocyte‐derived stimulated macrophages in the aged lung (Canan et al. 2014). For circulating‐derived macrophages (MDM, or BMDM), divergent results exist depending on experimental systems and detection methods. For instance, in a murine muscle injury model, BMDMs from aged mice exhibited significantly reduced phagocytosis of IgG‐coated beads (Fix et al. 2021). Similarly, BMDMs from aged mice showed impaired phagocytosis of CFSE‐labeled apoptotic cells (Peradinovic et al. 2023). Conversely, phagocytosis of fluorescent microspheres (Linehan et al. 2014) or bacteria (Hachim et al. 2017) showed no significant age‐related differences in other studies. A recent study using human peripheral blood monocyte‐derived macrophages reported impaired phagocytic capacity in cells from older donors (Moss et al. 2024); however, the exclusive reliance on image‐based quantification raises concerns about the reliability of this conclusion. Furthermore, another image‐based assay showed no difference in phagocytosis by IFN‐γ/LPS‐polarized mouse BMDMs from 8‐week‐old versus 18‐month‐old mice (Hachim et al. 2017).

Macrophage functionality integrates their ontogeny, local microenvironment, and encountered pathogenic stimuli. Crucially, the inability to distinguish monocyte‐derived cells from bona fide tissue‐resident macrophages—combined with oversimplified phagocytosis assays—undermines the robustness of existing studies. Furthermore, the lack of mechanistic exploration fails to provide intervention strategies targeting age‐related phagocytic decline.

The rearrangement of the cytoskeleton is the structural basis for phagocytosis. The actin network is a complex system with variable shapes, sizes, and filamentous architectures, including branched actin filaments at the leading edge and sites of endocytosis, tightly packed linear bundles in filopodia, and antiparallel arrays in muscle sarcomeres, stress fibers, etc. (Goode et al. 2023). The turnover rates of actin filaments are usually very fast at the leading edge and sites of endocytosis (Lacy et al. 2019; Watanabe and Mitchison 2002), and relatively slow in stereocilia and sarcomeres (Narayanan et al. 2015; Rzadzinska et al. 2004), which fit the physiological function very well. It has been reported that dense cortical actin cytoskeleton beneath cell membrane prevents phagocytosis, while parts of the cell where Rho‐family GTPase was inhibited and with thinner cortical actin filaments were better able to engage particles (Freeman et al. 2018). Whether and how aging affects the macrophage cytoskeleton, especially actin filament‐controlled phagocytosis, remains unknown.

Here, by comparing MDMs from young and aged human donors, we discovered that aged macrophages significantly upregulate extracellular matrix (ECM) genes, notably collagens, in response to LPS. We demonstrate that collagen overproduction impedes phagocytosis by direct binding to F‐actin and inhibits actin filament turnover. Crucially, this defect is driven by mitochondrial ROS (mtROS), as scavenging mtROS restores phagocytic capacity by normalizing collagen expression and rescuing actin dynamics. Our findings unveil a targetable axis linking mitochondrial redox imbalance and collagen‐controlled actin system dynamics in aged macrophages.

## 2 Results

### 2.1 Phagocytic Impairment in Aged MDMs Across Species

**FIGURE 1:**
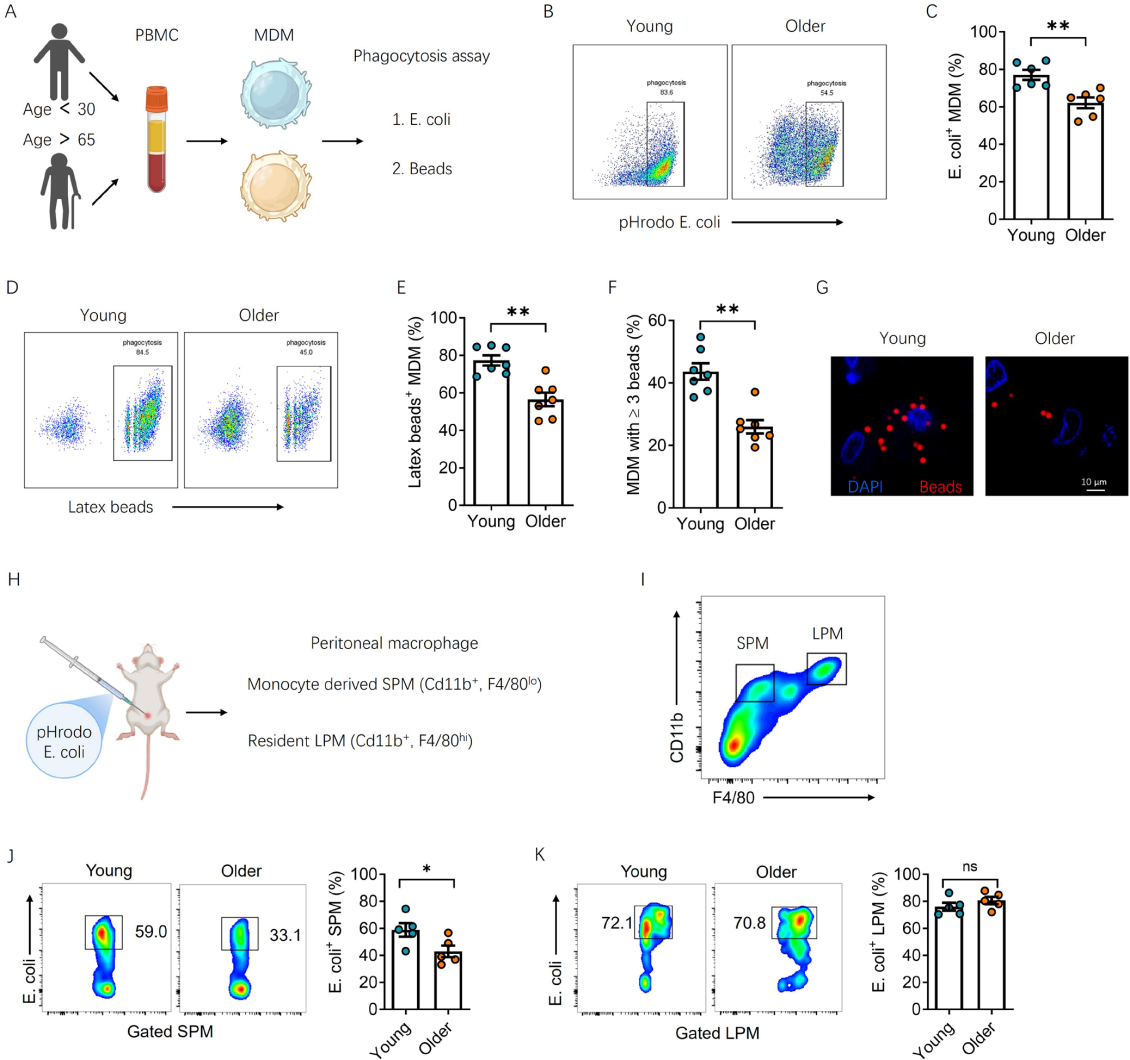
Aging dampens phagocytic activity in monocyte derived macrophages. (A) Experimental design of human MDMs phagocytic activity assay. (B, C) Flow cytometry quantification of pHrodo‐*E. coli* phagocytosis in MDMs from young and elderly donors (*n* = 6 each). (D, E) Flow cytometry quantification of fluorescence‐labeled latex beads phagocytosis in MDMs from young and elderly donors (*n* = 7 each). (F) Percentage of MDMs phagocytosing 3 or more beads in both group (*n* = 7 each). (G) Representative confocal microscopy images showing intracellular localization of phagocytosed fluorescence‐latex beads in hMDMs. (H) Experimental design of peritoneal macrophage phagocytic activity assay. Peritoneal macrophages were harvested 4 h post‐injection of pHrodo‐ *E. coli* . (I) Example of large peritoneal macrophages (LPMs, CD11b+, F4/80hi) and small peritoneal macrophages (SPMs, CD11b+, F4/80lo) in flow cytometry assay. (J) Flow cytometry quantification of pHrodo‐*E. coli* containing SPM in young and old mice (*n* = 5 each). (J) Quantification of pHrodo‐*E. coli* containing LPM in young and old mice (*n* = 5 each). All data are mean ± SEM. Two‐tailed, unpaired Mann–Whitney–Wilcoxon rank test (C, E, F, J, K): *p < 0.05, **p < 0.01.

MDMs were derived in vitro from peripheral blood mononuclear cells (PBMCs) obtained from two gender‐balanced groups (Figure 1A): aged 18–30 years (young cohort) or > 65 years (older cohort). Following activation with LPS, the phagocytic capacity of MDMs was assessed using fluorescent *E. coli* . Significantly, over 75% of MDMs from the young group were capable of phagocytosing *E. coli* , whereas this percentage dropped to 60% in the older group (Figure 1B,C). A similar decrease was observed when assessing the phagocytosis of opsonized fluorescent beads by MDMs from older humans (Figure 1D,E). Notably, the percentage of cells phagocytosing more than 3 beads—which we defined as “super‐phagocytes”—decreased more than twofold in the older group (Figure 1F). Using confocal microscopy, intracellular localization of beads was observed indicating phagocytosis, not simply tethering on macrophage surface (Figure 1G). Given that macrophage phagocytosis can be triggered by distinct mechanisms, we performed additional phagocytosis assays using latex beads under three different conditions: unopsonized, opsonized with human serum (complement‐mediated), and opsonized with human IgG (Fc‐mediated). Interestingly, the age‐related impairment in phagocytosis persisted across all three conditions (Figure S1B). We reanalyzed the data from a recently published single cell RNAseq database of healthy human blood cells (Terekhova et al. 2023), and found that myeloid cells remain relatively static throughout aging, with no age‐associated proportional change between classical and nonclassical monocyte (Figure S1). Therefore, the difference between MDMs from young and aged subjects is likely not due to differences in monocyte subtypes.

To assess phagocytic function in vivo, we injected fluorescent *E. coli* into the peritoneal cavity of young and old mice. Peritoneal macrophages were harvested 2 h post‐injection and analyzed by flow cytometry. These macrophages can be categorized based on developmental origins into large peritoneal macrophages (LPMs) and small peritoneal macrophages (SPMs), identifiable by CD11b and F4/80 staining (Figure 1H,I). LPMs originate from embryonic precursors and represent the tissue‐resident population. In contrast, SPMs are primarily monocyte‐derived and expand substantially in response to infectious stimuli (Ardavin et al. 2023). While overall phagocytic activity by the total macrophage population did not differ significantly between young and old mice, we observed a specific defect in SPMs from mice older than 20 months. SPMs from older mice phagocytosed significantly fewer bacteria compared to their young counterparts (Figure 1J). In contrast, the phagocytic capacity of LPMs was comparable between young and older groups (Figure 1K). These findings demonstrate an age‐associated phagocytic impairment specifically in monocyte‐derived macrophages (SPMs) within the peritoneal cavity, mirroring the defect observed in human MDMs in vitro, suggesting it may represent a conserved risk factor for impaired infection clearance in aging.

### 2.2 Aged MDMs Upregulates Collagen Production

**FIGURE 2:**
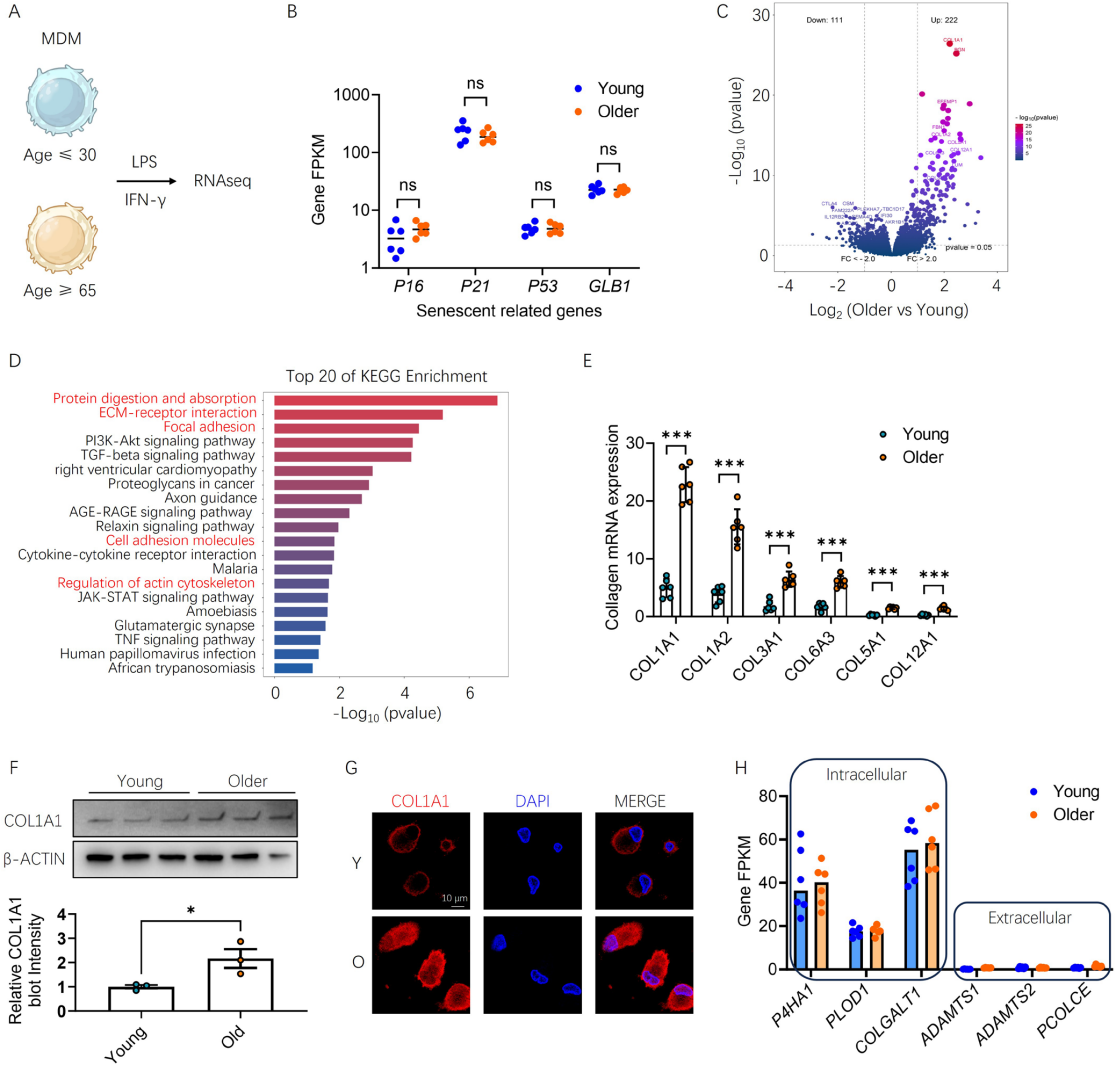
Aged MDMs upregulate collagen family genes expression. (A–E) Transcriptomic analysis of hMDMs. Schematic diagram of RNA sequencing analysis in hMDMs. (A; *n* = 6 each). Senescence marker gene expression in young and aged groups (B). Volcano plot showing the gene expression fold change and *p*‐value in young and older groups (C). Top 20 KEGG pathways in DEGs (D). Collagen family genes expression in MDMs from young and older groups (E). (F, G) COL1A1 protein was detected by western‐blot (F) and immunofluorescence staining (G) in young and older hMDMs. Representative confocal images of COL1A1 staining (red) (G). Scale bar, 10 μm. (H) Gene expression of key enzymes involved in collagen synthesis and maturation processes. All data are mean ± SEM. Two‐tailed, unpaired Mann–Whitney–Wilcoxon rank test (B, E, H): *p < 0.05, ***p < 0.001.

Having identified a significant phagocytic impairment in MDMs from older subjects, we sought to elucidate the underlying mechanism through RNA sequencing analysis (Figure 2A). Intriguingly, canonical senescence marker genes, including P16, P21, P53, and GLB1, showed no differential expression between young and older groups (Figure 2B). Also, GSEA analysis revealed no difference in phagocytosis, endocytosis, or macrophage migration (Figure S2A–C). And we confirmed that the mRNA level of key phagocytic receptors was not different between young and older hMDMs (Figure S2D). However, an unexpected striking transcriptional signature emerged in aged MDMs, characterized by the upregulation of extracellular matrix (ECM)‐related genes, particularly members of the collagen family and fibronectin (Figure 2C). KEGG pathway analysis of differentially expressed genes revealed that upregulated genes in aged MDMs were predominantly ECM‐associated, including “Protein digestion and absorption,” “ECM‐receptor interaction,” and “Focal adhesion”, importantly, these genes were linked to biological processes such as “cell adhesion” and “regulation of actin cytoskeleton”—pathways critically involved in phagocytic activity (Figure 2D). These findings suggest that aging transcriptionally reprograms macrophages by altering ECM protein expression and cytoskeletal organization, potentially contributing to their impaired phagocytic capacity.

Among the top enriched genes in aged MDMs, we focused on the collagen family, the most abundant ECM proteins in the body. While collagen is primarily synthesized by fibroblasts, its production in macrophages and its functional implications remain poorly understood. Among the different collagen subtypes, COL1A1 exhibited the highest expression in macrophages (Figure 2E) and was significantly upregulated in MDMs from the older group. To understand whether collagen genes are also expressed in monocytes, we analyzed published RNA‐seq data (GSE243175) and found that monocytes do not express COL1A1 and COL1A2 at all; these genes are expressed only after induction into macrophages (Figure S2E,F). We confirmed COL1A1 overproduction at the protein level via Western blot (Figure 2F), also, immunofluorescence staining showed more abundant cytoplasmic COL1A1 in aged MDMs (Figure 2G).

Collagen synthesis and maturation involve several critical post‐translational modifications and proteolytic processing steps (Onursal et al. 2021). We assessed the expression of key enzymes involved in these processes, including P4HA1 (catalyzing prolyl‐4‐hydroxylation), PLOD1 (mediating lysine hydroxylation), COLGALT1 (catalyzing subsequent glycosylation). However, none of these enzymes showed differential expression between young and aged MDMs (Figure 2H). Intriguingly, macrophages exhibited minimal expression of extracellular collagen‐processing enzymes such as ADAMTS1/2 and PCOLCE (Figure 2H), suggesting that macrophages may not produce fully mature, secreted collagen fibrils. Using Western blot, we can easily detect COL1A1 in the cell pellet but not in the culture supernatant (Figure S2G), confirming that in vitro‐induced M1 macrophages do not secrete substantial amounts of mature collagen I. These data collectively indicate that aged macrophages upregulate collagen synthesis, particularly COL1A1, but likely retain it intracellularly or in a non‐fibrillar form due to insufficient processing machinery.

### 2.3 Knockdown and Overexpression of COL1A1 Regulate Phagocytosis

**FIGURE 3:**
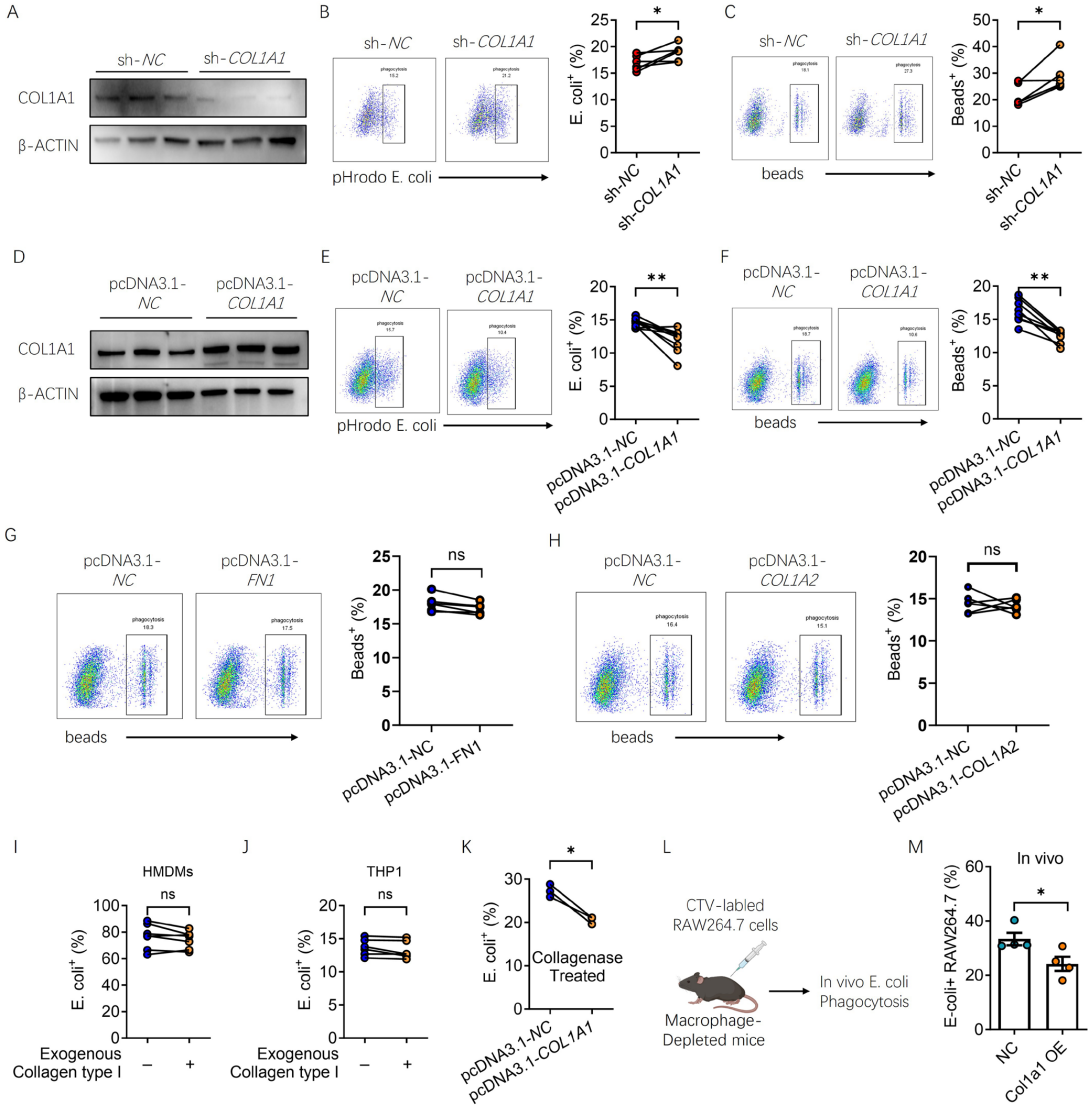
Manipulation of type I collagen expression regulates macrophage phagocytosis. (A) Knocking down (KD) of COL1A1 expression by sh‐COL1A1 in macrophages were assessed by WB. (B, C) Flow cytometric measurement of phagocytosis using pHrodo *E*. *coli* BioParticles (B; *n* = 6 each) and fluorescence‐labeled latex beads (C; *n* = 6 each). (D) Overexpression (OE) of COL1A1 by pcDNA3.1‐COL1A1 in macrophages were assessed by WB. (E, F) Flow cytometric measurement of phagocytosis using pHrodo *E*. *coli* BioParticles (E; *n* = 9 each) and fluorescence‐labeled latex beads (F; *n* = 8 each). (G, H) Flow cytometric measurement of phagocytosis using fluorescence‐labeled latex beads after FN1 and COL1A2 overexpression in macrophages (both *n* = 6 each) (I, J) Flow cytometry quantification of pHrodo‐*E*. *coli* phagocytosis in hMDMs (G; *n* = 7 each) and THP‐1 cell lines (H; *n* = 6 each) after exogenous collagen pre‐treatment. (K) Flow cytometry quantification of pHrodo‐*E*. *coli* phagocytosis in macrophages transfected with either NC plasmid or pcDNA3.1‐COL1A1 overexpression plasmid, followed by collagenase treatment (1 mg/mL for 2 h; *n* = 3 each). (L) Experimental design of in vivo phagocytosis assay. Simply, mice were treated with clodronate liposomes to deplete macrophages, and then RAW 264.7 cells transfected with indicated plasmid (NC or Col1a1 overexpression) were adoptively transferred into mice peritoneal to perform phagocytosis of GFP‐*E*. *coli*. (M) Flow cytometry quantification of GFP‐*E*. *coli* phagocytosis in RAW 264.7 cells (*n* = 4 each). All data are mean ± SEM. Two‐tailed, paired Student's *t*‐test (B, C, E, F, G, H, I), two‐tailed, unpaired Mann–Whitney–Wilcoxon rank test (K): *p < 0.05, **p < 0.01.

To investigate whether the overexpression of collagen family genes is causally linked to the phagocytic defect in aged MDMs, we modulated COL1A1 expression in the human monocytic cell line THP1 by electroporating plasmids for either overexpression or shRNA‐mediated knockdown. Following transfection, macrophage differentiation was induced with PMA and THP‐1 derived macrophages were then activated with LPS/IFN‐γ. We confirmed that shRNA targeting COL1A1 achieved efficient knockdown (Figure 3A). Functionally, knockdown of COL1A1 significantly enhanced macrophage phagocytosis of bacteria (Figure 3B) and beads (Figure 3C). Conversely, overexpression of COL1A1 markedly impaired phagocytic function (Figure 3D–F). We extended our investigation to other prominently upregulated ECM genes. Notably, overexpression of Fibronectin1 (FN1) or COL1A2 failed to recapitulate the phagocytic impairment observed with COL1A1 (Figure 3G,H), indicating a specific and non‐redundant role for COL1A1 in regulating macrophage phagocytic capacity. To clarify whether phagocytic impairment specifically requires collagen overproduction by macrophages or can also be induced by extracellular collagen, we pretreated both HMDMs and THP1‐derived macrophages with soluble human type I collagen for 2 h prior to *E. coli* phagocytosis assays. Intriguingly, exogenous collagen pretreatment did not affect phagocytosis (Figure 3I,J). Also, digesting extracellular collagen with collagenase cannot abrogate the inhibition of phagocytosis upon COL1A1 overexpression (Figure 3K). These data indicate that the anti‐phagocytic effect stems from macrophage‐intrinsic collagen production and cannot be substituted by extracellular collagen, suggesting an intracellular mechanism of action.

Given the technical challenge of specifically overexpressing collagen in monocyte‐derived macrophages (MDMs) in vivo, we employed an indirect approach to investigate whether collagen overproduction could impact phagocytic behavior within a mouse model. We depleted endogenous macrophages in mice using clodronate liposomes and adoptively transferred RAW 264.7 murine macrophage cells (engineered to overexpress Col1a1) into the peritoneal cavity of these mice (Figure 3L). We then assessed bacterial phagocytosis of transferred macrophages in vivo. Strikingly, RAW 264.7 cells overexpressing Col1a1 phagocytosed significantly fewer fluorescence‐labeled *E. coli* compared to the control group within the peritoneal cavity (Figure 3M). Collectively, these results demonstrate that modulating collagen gene expression within macrophages directly regulates their phagocytic activity, and crucially, this effect is independent of exogenous collagen.

### 2.4 Collagen Binding Slows Down F‐ACTIN Turnover Rate

**FIGURE 4:**
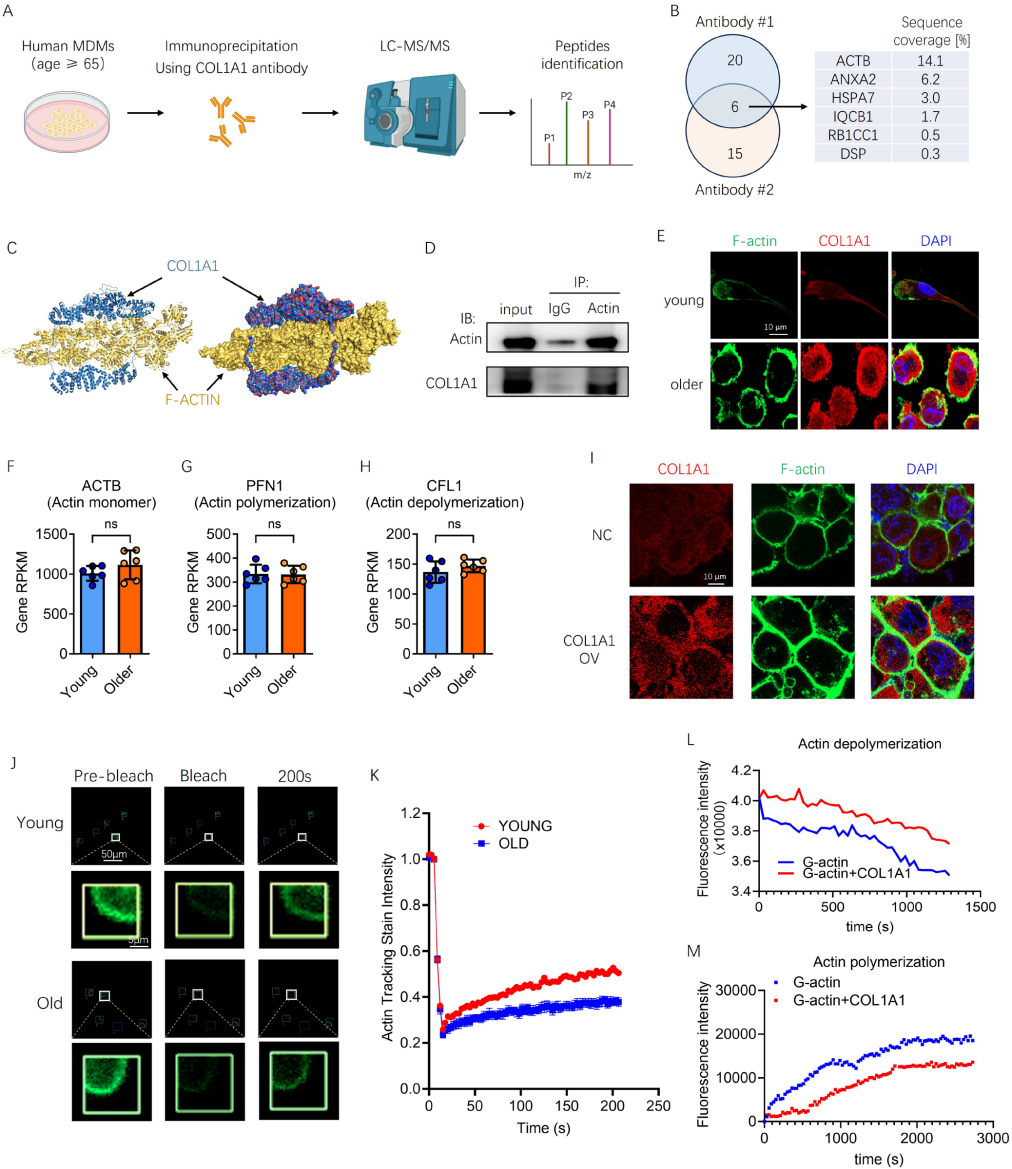
Collagen binding inhibits F‐Actin turnover rate. (A) Schematic diagram of Immunoprecipitation‐Mass Spectrometry (IP‐MS) analysis. (B) Venn plot showing identified proteins in experiments using two different COL1A1 monoclonal antibodies. (C) Conformational diagram of F‐Actin (G‐Actin octamer) and COL1A1 molecular docking via AlphaFold. (D) Confirmation of COL1A1‐Actin binding in hMDMs by Co‐IP and western‐blot. (E) Representative confocal image of F‐Actin (green) and COL1A1 (red) from young and aged hMDMs. Scale bar, 10 μm. (F–H) Gene expression levels of key genes involved in regulating F‐Actin turnover from RNA sequencing analysis. (I) Representative confocal image of F‐Actin (green) and COL1A1 (red) in THP‐1 derived macrophages transfected with either NC plasmid or pcDNA3.1‐COL1A1 plasmid (*n* = 6 each). (J) The rate of Actin turnover was analyzed by FRAP. Images before and after (200 s) bleaching are shown. (K) The recovery curve of fluorescent intensity after bleaching. (L, M) Kinetics of depolymerization (L) and polymerization (M) of Actin filaments in the presence of COL1A1. All data are mean ± SEM. Two‐tailed, unpaired Mann–Whitney–Wilcoxon rank test (F–H): *p < 0.05, **p < 0.01, ***p < 0.001.

To elucidate the mechanism by which collagen overexpression impairs phagocytosis, we investigated whether collagen affects essential phagocytic components, such as phagocytic receptors or the cytoskeletal machinery. We identified potential collagen‐interacting proteins using immunoprecipitation coupled with mass spectrometry (IP‐MS) with two distinct monoclonal antibodies targeting human COL1A1 (Figure 4A). Six proteins were consistently identified with both antibodies. Among these, ACTB (β‐actin) and ANXA2 (Annexin A2) exhibited the highest sequence coverage (Figure 4B). ACTB is the primary constituent of filamentous actin (F‐actin), which is intimately associated with the plasma membrane of phagocytic cells (Mylvaganam et al. 2021). As direct binding between collagen and either F‐actin or monomeric G‐actin has not been previously reported, we employed AlphaFold to model the interaction. Using a short F‐actin fragment modeled as an ACTB octamer, we predicted its binding with COL1A1 (Figure 4C). The model suggests COL1A1 can bind simultaneously to two chains of F‐actin, with interaction energies (ΔiG) below −10 kcal/mol for binding to three ACTB monomers (Figure S3), indicating the potential for a stable complex, and we also confirmed the binding between ACTB and COL1A1 via immunoprecipitation and immunoblotting (Figure 4D).

The actin cytoskeleton plays a pivotal role in phagocytosis, a fact we confirmed by treating cells with cytochalasin D (CytoD), which severely impaired phagocytic activity—particularly against larger fluorescent beads (Figure S4), suggesting a stronger dependence on actin turnover compared to bacterial uptake. The structure and turnover rate of F‐actin critically regulate macrophage phagocytic capacity. For instance, increasing cortical actin thickness using the Rho activator CN03 significantly inhibits ruffle formation in macrophages (Freeman et al. 2018). In aged MDMs, cortical F‐actin staining is much stronger (Figure 4E), however, proteins regulating F‐actin polymerization, branching, and depolymerization are not affected by age (Figure 4F–H). We therefore hypothesized that intracellular collagen in macrophages might impede F‐actin turnover directly, thereby affecting phagocytic behavior. To test this, we overexpressed COL1A1 in THP‐1‐derived macrophages and observed a significant increase in F‐actin thickness beneath the plasma membrane (Figure 4I). To directly visualize the spatial relationship between nascent COL1A1 and the cytoskeleton, we overexpressed GFP‐COL1A1 in THP‐1‐derived macrophages and stained for F‐actin with phalloidin. Confocal microscopy showed that COL1A1 specifically accumulated in the cortical cytoskeletal compartment, where it directly overlapped with F‐actin filaments beneath the plasma membrane (Figure S4D). This spatial juxtaposition indicates a close physical association between intracellular COL1A1 and the submembrane actin network.

To investigate the dynamic remodeling capacity of the macrophage cytoskeleton during aging, we performed fluorescence recovery after photobleaching (FRAP) with an F‐actin probe (Figure 4J). Quantitative analysis revealed a significant impairment in actin turnover kinetics in macrophages derived from aged donors (Figure 4K). Furthermore, F‐actin depolymerization rate assays revealed that the addition of exogenous collagen slowed down actin filament disassembly (Figure 4L), also, G‐actin polymerization into F‐actin is disrupted by collagen (Figure 4M), indicating an overall inhibitory effect on actin system turnover. Intriguingly, ANXA2, another collagen binding protein identified in mass spectrometry (Figure 4B), binds more strongly with ACTB upon COL1A1 overexpression (Figure S4), suggesting a potential secondary mechanism since ANXA2 is known to affect actin filament turnover (Hayes et al. 2006). These results collectively indicate that macrophage‐derived collagen stabilizes the cortical actin network through both direct interaction with F‐actin and potentially indirect modulation via ANXA2, ultimately restricting membrane plasticity and impairing phagocytic function.

### 2.5 Mitochondria ROS Drive Collagen Overproduction in Aged Macrophages

Although macrophages were historically not considered major producers of collagen, emerging evidence indicates that they express nearly all known collagen mRNAs (Schnoor et al. 2008) and contribute to tissue fibrosis in the heart and lung (Tsitoura et al. 2021; Simoes et al. 2020), However, the regulatory mechanisms controlling collagen production in macrophages remain unclear.

**FIGURE 5:**
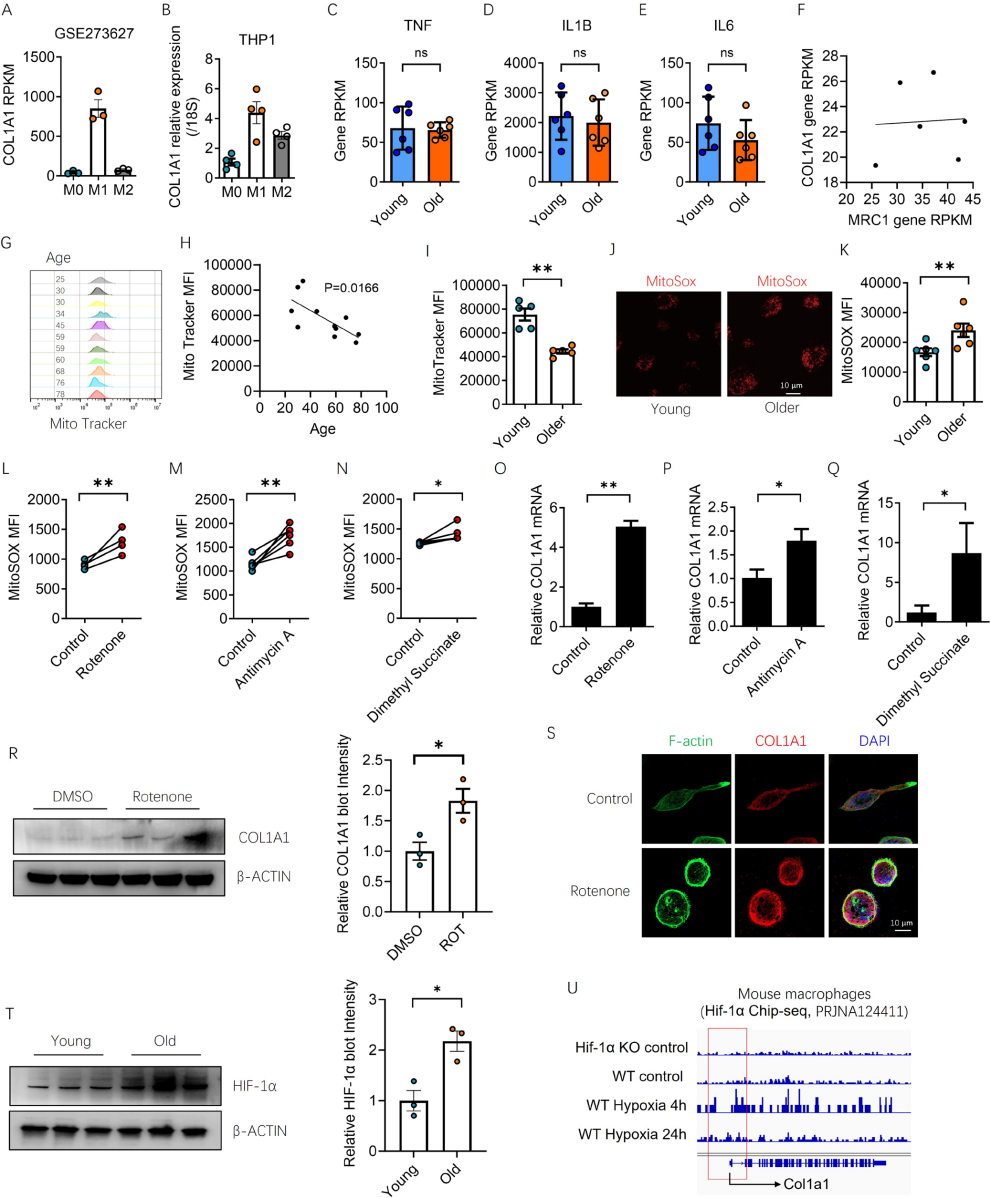
Excessive mtROS in aged MDMs drives collagen overproduction. (A) COL1A1 gene expression in M0, M1 and M2 polarized hMDMs from published RNA‐seq data (GSE273627). (B) qRT‐PCR analysis of COL1A1 mRNA expression in M0, M1 and M2 of THP‐1 derived macrophages (*n* = 4 each). (C–E) Transcriptome for inflammatory markers, TNF, IL1B, IL6, in young and aged groups as indicated (*n* = 6 each). (F) Correlation between COL1A1 and MRC1 gene expression from RNA‐seq analysis in hMDMs (*n* = 6). (G, H) Correlation between hMDMs mitochondrial membrane potential (ΔΨm) (MitoTrackerRed staining MFI) and age (*n* = 10). (I) Quantification of MitoTrackerRed MFI values between hMDMs from young and older group (*n* = 5 each). (J, K) Detection of mtROS via MitoSOX staining. Representative Confocal images (J), scale bar, 10 μm, flow cytometry quantification (K). (L–N), Induction of mtROS production by rotenone (1 μM), antimycin A (10 μM), and dimethyl succinate (1 mM) for 24 h. MitoSOX MFI was measured by flow cytometry (L, *n* = 4; M, *n* = 6; N, *n* = 6). (O–Q). qRT‐PCR analysis of COL1A1 mRNA expression in each group (*n* = 3 each). (R) Western‐blot detection of increased COL1A1 protein level in Rotenone treated cells. (S) Representative confocal images of F‐Actin (green) and COL1A1 (red) in cells treated with Rotenone (1 μM) for 24 h. (T) Western‐blot detection of HIF‐1α in young and old HMDMs. (U) Analysis of HIF‐1α binding with *COL1A1* gene in published macrophage Chip‐seq data (PRJNA124411). All data are mean ± SEM. Two‐tailed, paired Student's *t*‐test (L–Q), two‐tailed, unpaired Mann–Whitney–Wilcoxon rank test (C–E, I, K, R, T): *p < 0.05, **p < 0.01.

We first analyzed published RNA‐seq data from the classical differentiation of monocytes into M0, M1, and M2 macrophages (Migliaccio et al. 2024). LPS/IFN‐γ‐induced M1 polarization led to a modest upregulation of collagen family genes (Figure 5A), though overall expression levels remained low, and we confirmed this expression mode using THP‐1 derived macrophages (Figure 5B), suggesting a potential link between collagen genes and TLR4 activation. However, when comparing gene expression profiles of MDMs from young and aged donors, we found no significant upregulation of TLR4 downstream inflammatory cytokines (e.g., TNF, IL1B, IL6) in aged MDMs (Figure 5C–E), indicating that elevated collagen expression in aging is unlikely to be a direct consequence of TLR4 signaling. Additionally, M2 polarization appeared unrelated to collagen upregulation, as the expression of the M2 marker MRC1 showed no correlation with COL1A1 levels in aged MDMs (Figure 5F).

Given that mitochondrial dysfunction and oxidative stress are hallmarks of aging (Lima et al. 2022), we hypothesized that mitochondrial‐derived stress signals might drive collagen overproduction in aged macrophages. To test this, we first measured mitochondrial activity using MitoTracker Red staining, reflecting mitochondrial membrane potential (MMP). Analysis of MDMs from 10 healthy donors revealed a clear age‐dependent decline in mitochondrial activity (Figure 5G,H). Flow cytometry and confocal microscopy comparison of samples from donors under 30 and over 70 years old confirmed that aged MDMs exhibited significantly lower MMP (Figure 5I) and stronger MitoSOX staining, indicating elevated mitochondrial ROS (mtROS) production (Figure 5J,K). The observed low MMP in aged MDMs makes it unlikely that elevated mtROS primarily originate from reverse electron transport (RET) at mitochondrial complex I (Tabata Fukushima et al. 2024). We therefore investigated alternative sources for this excess mtROS. Gene expression analysis revealed a positive correlation between collagen gene expression and genes encoding components of the mitochondrial electron transport chain (ETC) and the mitochondrial fusion protein MFN2 (Figure S5A–F). MFN2 has been reported as critical for mitochondrial fusion and mtROS production in macrophages (Casey et al. 2025). In contrast, expression levels of other mitochondrial housekeeping genes (e.g., TOMM20, TOMM40, HSP60) or the fission protein DRP1 showed no correlation with COL1A1 expression (Figure S5G–J). These data collectively suggest that the overproduction of mtROS in aged MDMs likely arises from alterations in mitochondrial remodeling (specifically fusion) and ETC activity following LPS stimulation.

To establish a direct causal link between mtROS and collagen expression, we employed two distinct strategies to induce mtROS production in young MDMs (Kline and Bowdish 2016) inhibition of the electron transport chain (ETC) to promote electron leakage (using rotenone or antimycin A) (Li et al. 2003; Chen et al. 2003) and (Gavazzi and Krause 2002) induction of mitochondrial hyperpolarization using cell‐permeable dimethyl succinate (Tabata Fukushima et al. 2024). Both interventions significantly increased mtROS levels, as measured by elevated MitoSOX staining (Figure 5L–N). Crucially, both strategies also robustly induced COL1A1 gene expression (Figure 5O–Q). In contrast, treatment with exogenous H_2_O_2_ failed to stimulate COL1A1 expression (Figure S6A–F), underscoring a specific role for mitochondrially derived ROS in regulating collagen gene transcription. Furthermore, the increase in collagen protein upon rotenone treatment was confirmed by Western blot analysis (Figure 5R). Notably, rotenone‐treated young MDMs also phenocopied the key cytoskeletal defect of aged cells, exhibiting significantly increased cortical F‐actin thickness (Figure 5S). Collectively, these results demonstrate that mtROS act as a primary driver inducing both collagen overproduction and the associated impairment in actin dynamics characteristic of aged macrophages.

Having established mtROS as an upstream inducer of collagen expression, we sought to identify the transcriptional mediator connecting mitochondrial stress to *COL1A1* gene activation. Given the known stabilization of Hypoxia‐Inducible Factor 1‐alpha (HIF‐1α) under conditions of mitochondrial dysfunction and oxidative stress (Huang et al. 2022), we found that aging leads to a significant accumulation of HIF‐1α protein in human MDMs (Figure 5T). According to the landmark genome‐wide HIF‐1α mapping study by Mole et al. (2009)., bona fide direct target genes controlled by the HIF cascade are strictly characterized by the clustering of core canonical Hypoxia Response Elements (HREs, 5′‐RCGTG‐3′) localized within the narrow window of ±2000 bp around the transcription start site (TSS). We systematically scanned the proximal promoters and early intronic structures of both human and mouse type I collagen, identifying 6 and 8 functional HRE motifs within the COL1A1 and Col1a1 genomic loci, respectively (Table 1).

**TABLE 1:** HRE in human and mouse Col1a1 gene promoter.

Species	Motif no.	Core motif	Strand	Position relative to TSS (bp)	Sequence context (5′ → 3′, motif bolder)	Region
Human (Col1a1)	1	GCGTG	Antisense (−)	−1870	agggggctgaggtgctgagatggca **GCGTG** cttgaacccaccaaagtcc	Distal promoter
	2	ACGTG	Sense (+)	−1685	gcccacggccagccggccagccg **ACGTG** ctccctccccttctgttcct	Distal promoter
	3	GCGTG	Sense (+)	−1570	ctgggggaggcgggactcccc **GCGTG** tttgcagctctggagcacccg	Distal promoter
	4	GCGTG	Sense (+)	271	gcaaggatactctatatcgcgcttg **GCGTG** ttggtcccgggggccgcgg	Exon 1
	5	ACGTG	Sense (+)	499	ggtgcagagtgaggaaagc **ACGTG** cgaagatgggatgggggcgccg	Intron 1
	6	ACGTG	Antisense (−)	1993	taatgctgctcccgtcggcagaggc **ACGTG** ctagagcgaccacccccagc	Intron 1
Mouse (Col1a1)	1	GCGTG	Sense (+)	−1959	caaagggtgagcagatcacacc **GCGTG** cgcgcgtgtgtgtgtgtgt	Distal promoter
	2	GCGTG	Sense (+)	−1953	agatcacaccgcgcgcgc **GCGTG** tgtgtgtgtgtgtgtgtgtgtgt	Distal promoter
	3	GCGTG	Antisense (−)	−1947	acacacacacacacacgcgc **GCGTG** tggtgatctgctaccctttg	Distal promoter
	4	GCGTG	Antisense (−)	−704	ccagaagaatgcttatgctta **GCGTG** atgagcagcagtgtgggcag	Proximal promoter
	5	ACGTG	Sense (+)	−153	ccacggccagcg **ACGTG** gctccctccccttctgttcccttggtc	Proximal promoter
	6	GCGTG	Sense (+)	69	ggagca ggaggc **GCGTG** gagtgaggccacgcatgagccgaagc	Exon 1
	7	ACGTG	Sense (+)	511	tggacacgggttggggcagagt **ACGTG** gaaaatgaaataaggcct	Intron 1
	8	ACGTG	Antisense (−)	1283	gagaggagagaaggggaaattcagccctgg **ACGTG** gtttgcgcgg	Intron 1

*Note:* Within proximal promoters and early intronic structures of both human and mouse type I collagen, there are 6 and 8 functional HRE motifs within the COL1A1 and Col1a1 genomic loci.

To determine whether HIF‐1α directly occupies these sites to regulate transcription, we analyzed a recently published, high‐quality HIF‐1α ChIP‐seq dataset specifically derived from macrophages (Isagawa et al. 2025). In WT untreated macrophages, the enrichment of HIF‐1α around the Col1a1 TSS was negligible, remaining indistinguishable from the background signal of the HIF‐1α KO group. Strikingly, hypoxia stimulation triggered a distinct, localized recruitment of HIF‐1αat both 4 h and 24 h (Figure 5U). Collectively, these data identified COL1A1 gene to be a redox‐sensitive gene which might receive transcriptional control by HIF‐1α.

### 2.6 Restoring Phagocytosis by Targeting mtROS and Collagen‐Actin Interaction

**FIGURE 6:**
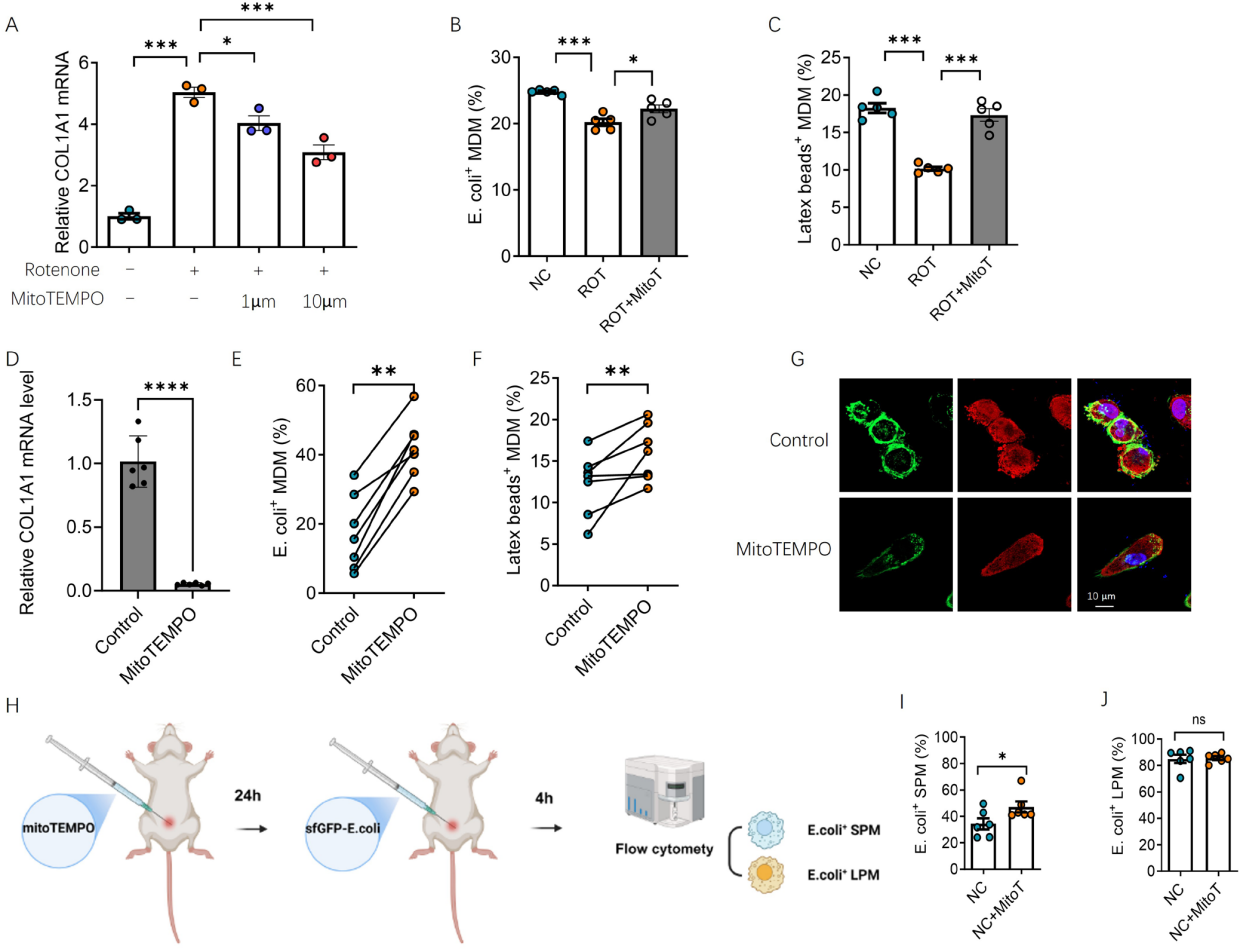
Scavenging mtROS rejuvenates macrophage phagocytic activity in vitro and in vivo. (A) qRT‐PCR analysis of COL1A1 mRNA expression in THP‐1 derived macrophage cells under three conditions: Untreated, rotenone‐treated (1 μM, 24 h), and MitoTEMPO‐rescued (1 or 10 μM, 2 h) (*n* = 3 each). (B, C) Flow cytometry quantification of phagocytosis using pHrodo‐ *E. coli* and fluorescence‐labeled latex beads in indicated treated group (B, *n* = 6 each; C, *n* = 5 each). (D) COL1A1 mRNA expression in aged hMDMs in presence or absence of MitoTEMPO (*n* = 6). (E, F) MitoTEMPO rescued phagocytic function against pHrodo *E. coli* and fluorescence‐labeled latex beads in aged hMDMs (E, *n* = 7 each; F, *n* = 7 each). (G) Representative confocal images of the expression and localization of F‐Actin (green) and COL1A1 (red) in aged hMDMs treated with MitoTEMPO. (H) Schematic diagram illustrating MitoTEMPO treatment (5 mg/kg, i.p.) and in vivo phagocytosis assay in old mice (> 20 months). (I, J) Flow cytometry assay of phagocytosed *E. coli* in SPM and LPM (*n* = 6 each). All data are mean ± SEM. One‐way ANOVA and post‐ANOVA, pair‐wise, two‐group comparisons conducted with Tukey's method (A–C), Two‐tailed, paired Student's *t*‐test (D–F), two‐tailed, unpaired Mann–Whitney–Wilcoxon rank test (I, J): *p < 0.05, **p < 0.01, ***p < 0.001.

To determine whether targeting mtROS‐collagen‐Actin axis could rescue the phagocytic defect in aged macrophages, firstly, we induced mtROS and phagocytic defect in THP1 derived macrophages using rotenone, then treated these cells with mitochondria‐targeted antioxidant MitoTEMPO (1 μM or 10 μM). MitoTEMPO dose‐dependently suppressed rotenone‐induced mtROS accumulation and prevented the upregulation of COL1A1 mRNA (Figure 6A). Importantly, MitoTEMPO treatment restored phagocytic function in rotenone‐treated cells, as evidenced by increased uptake of fluorescent *E. coli* (Figure 6B) and opsonized beads (Figure 6C). Next, we tested whether MitoTEMPO could rejuvenate phagocytosis in aged MDMs. Treatment with 10 μM MitoTEMPO significantly reduced COL1A1 expression (Figure 6D) and enhanced the phagocytic capacity of aged MDMs for both *E. coli* (Figure 6E) and beads (Figure 6F). Confocal microscopy confirmed that MitoTEMPO reduced intracellular COL1A1 levels and diminished cortical F‐actin thickness beneath the plasma membrane (Figure 6G), suggesting restoration of actin dynamics.

To validate these findings in vivo, we intraperitoneally administered MitoTEMPO to aged mice 24 h before injecting fluorescent *E. coli* (Figure 6H). Analysis of peritoneal macrophages 4 h post‐infection revealed that MitoTEMPO pretreatment significantly enhanced bacterial phagocytosis by small peritoneal macrophages (SPMs) (Figure 6I), while large peritoneal macrophages (LPMs) remained unaffected (Figure 6J). Importantly, flow cytometry also revealed that Col1a1 expression in SPMs of the MitoTEMPO‐treated group was significantly lower than that of the control group, accompanied by decreased F‐actin staining, suggesting in vivo efficacy of mitoTEMPO targeting the signaling axis (Figure S6F,G). These results demonstrate that mtROS scavenging selectively improves phagocytic function in monocyte‐derived macrophages (SPMs), further supporting the critical role of mitochondrial redox balance in age‐related immune decline.

Together, these findings establish that targeting the mtROS‐collagen‐actin axis can effectively restore macrophage phagocytosis during aging, offering a potential therapeutic strategy to bolster antimicrobial immunity in the elderly.

## 3 Discussion

Phagocytosis is central to macrophage function in two critical ways: it serves as the primary mechanism for eliminating invading pathogens and apoptotic cells, and it actively modulates macrophage functional states. For instance, phagocytosis following initial activation can drive macrophages toward an anti‐inflammatory phenotype, facilitating tissue repair and inflammation resolution (Watanabe et al. 2019). However, the impact of aging on macrophage phagocytosis has remained controversial, largely due to the developmental heterogeneity and functional plasticity of macrophage populations (Guan et al. 2025). For example, in a sterile skin inflammation model, aged human macrophages display defective phagocytosis and delayed inflammation resolution (De Maeyer et al. 2020) due to reduced TIM4 expression—a key receptor for phosphatidylserine on apoptotic cells (Flannagan et al. 2014). Furthermore, aging reduces the expression of the scavenging receptor CD204 in alveolar macrophages (key cells for maintaining lung homeostasis), causing neutrophil retention during lung infection and exacerbating lung injury (Wong et al. 2017). Notably, LPS‐stimulated aged MDMs in our system showed no deficiency in TIM4 or other phagocytic receptors such as CD204, underscoring the existence of subset‐specific aging mechanisms.

Our study uncovers a mitochondrial dysfunction‐driven pathway underlying macrophage aging, characterized by excessive mtROS production and consequent upregulation of collagen family genes. This pathway leads to excessive stabilization of the F‐actin cytoskeleton in aged MDMs, impairing their ability to dynamically remodel the actin network during phagocytosis. Importantly, we demonstrate that both genetic modulation of collagen expression and pharmacological scavenging of mtROS can restore phagocytic function in aged macrophages, both in vitro and in vivo. Crucially, we found that circulating monocytes in the bloodstream do not express COL1A1 at baseline. Instead, during their differentiation into macrophages, stimulation with bacterial components (LPS) and pro‐inflammatory cytokines (IFN‐γ) significantly upregulates collagen‐related genes. This indicates that the mtROS‐collagen axis we identified is primarily activated during inflammatory macrophage differentiation. Our in vivo findings further support this, showing that aging predominantly impairs the phagocytic capacity of small peritoneal macrophages (SPMs)—which are monocyte‐derived—rather than tissue‐resident populations. This highlights the critical role of this signaling axis in restraining monocyte maturation and the acquisition of effector phagocytic functions during aging.

Macrophages exhibit dynamic morphology even at baseline, enabling environmental surveillance (Hanna et al. 2015; Nimmerjahn et al. 2005). During phagocytosis, cytoskeletal dynamics become paramount—successful particle internalization requires an exquisite balance between actin polymerization and depolymerization, orchestrated by actin‐binding proteins (ABPs) (Rougerie et al. 2013; Diakonova et al. 2002). Here, we identify collagen as a novel macrophage ABP that regulates cortical F‐actin turnover, which is critical for cell membrane flexibility during phagocytosis. Using in vitro disassembly assays, we demonstrate collagen‐mediated stabilization of F‐actin networks. Furthermore, we reveal that the collagen production pattern in macrophages is characterized by the absence of key extracellular processing enzymes, highlighting its likely intracellular function. Strikingly, COL1A1 overexpression in young MDMs phenocopies the phagocytic defect of aged MDMs, particularly for larger targets like latex beads. This size‐dependent impairment suggests F‐actin turnover is especially critical for engulfing substantial particles, where significant membrane deformation demands greater cytoskeletal flexibility.

Because this cytoskeletal stiffening fundamentally alters macrophage structural plasticity, the resulting phagocytic impairment is not restricted to a specific receptor pathway. Our data demonstrate a consistent age‐related decline in phagocytic capacity across unopsonized, complement‐opsonized, and IgG‐opsonized targets. Whether particle internalization is driven by extensive actin‐rich protrusions or local receptor‐mediated sinking (Allen and Aderem 1996; Caron and Hall 1998), the process demands rapid and dynamic remodeling of the cortical actin network. Thus, the intracellular accumulation of collagen imposes a global structural constraint on F‐actin turnover rather than a localized defect in specific opsonic or scavenging receptors.

Furthermore, an important distinction must be made regarding the fate of this macrophage‐produced collagen. While macrophages are known to contribute to extracellular matrix (ECM) deposition during tissue repair and scar formation (Simoes et al. 2020), our data indicate that in a highly pro‐inflammatory microenvironment, the synthesized collagen remains predominantly intracellular. This is supported by our observation that M1‐polarized macrophages lack critical extracellular collagen‐processing enzymes, such as ADAMTS1/2 (Tang 2001), preventing the maturation and secretion of fibrillar collagen. Consequently, the immediate pathological impact of collagen in aged pro‐inflammatory macrophages is cell‐intrinsic—paralyzing their own cytoskeletal machinery—rather than actively driving tissue fibrosis through ECM secretion. However, the long‐term fate of these collagen‐loaded macrophages in aging tissues remains an important question, as their defective clearance of pathogens and apoptotic cells could indirectly promote chronic inflammation and subsequent fibrotic outcomes.

The precise reason why aged MDMs utilize mtROS signaling to downregulate phagocytosis warrants further investigation. It may represent a negative feedback mechanism, as metabolic decline is a hallmark of cellular aging (Palmer and Jensen 2022). For macrophages, the breakdown and utilization of large amounts of extracellular particles could impose a substantial metabolic burden, particularly during the transitional phase from monocyte to macrophage when downstream machinery like lysosomes and related enzymes may not be fully primed. Importantly, in aged mice, scavenging mtROS with MitoTEMPO significantly enhanced bacterial phagocytosis by peritoneal SPMs. This demonstrates the therapeutic potential of targeting the mtROS‐collagen‐phagocytosis axis. Especially in elderly patients prone to recurrent infections, interventions aimed at improving mitochondrial homeostasis—such as enhancing mitophagy (Cen et al. 2020) to bolster monocyte adaptation to metabolic challenges—could enable rapid pathogen control during early infection, preventing protracted inflammation.

## 4 Materials and Methods

### 4.1 Mice

Male C57BL/6J mice were obtained from the Experimental Animal Center of Hangzhou Medical College (Hangzhou, China). Young (8‐week‐old) and aged (20‐month‐old) mice were co‐housed in a specified pathogen‐free facility under a 12‐h light/dark cycle with ad libitum access to food and water. For all mouse studies, ≥ 4 mice were used for each experimental condition. All animal procedures were approved by the Animal Experiment Ethics Committee of the First Affiliated Hospital, Zhejiang University School of Medicine (Approval No. 2024‐1740).

### 4.2 Human Subjects

Venous blood samples were collected from healthy sex‐balanced donors into 3.0 mL EDTA‐K_2_ anticoagulant vacuum tubes (Becton Dickinson, USA) at the First Affiliated Hospital, Zhejiang University School of Medicine. Written informed consent was obtained from all participants, and the study protocol was approved by the hospital's Ethics Committee (Approval No. IIT20250005A). Exclusion criteria comprised: current or prior cancer diagnosis, uncontrolled medical conditions, or chronic inflammatory syndromes. Information about all human subjects is listed in Table S3. All procedures adhered to local ethics regulations.

### 4.3 Macrophage Preparation and Culture

Human peripheral blood mononuclear cells (PBMCs) were isolated via Lymphoprep density gradient centrifugation (STEMCELL Technologies, Canada, Catalog no. 7861). Monocytes were enriched by 2‐h adhesion in serum‐free RPMI 1640 medium (MeilunBio, China, Catalog no. MA0215), followed by culture in RPMI 1640 supplemented with 10% FBS (Gibco, USA, Catalog no. C0235) and 1% penicillin‐streptomycin (MeilunBio, China, Catalog no. MA0110). Differentiation into macrophages was induced using 20 ng/mL recombinant human M‐CSF (BioLegend, USA, Catalog no. 574804) for 5–7 days.

THP‐1 cells were maintained in RPMI 1640 with 10% FBS and 1% penicillin–streptomycin. Macrophage differentiation was triggered with 100 ng/mL PMA (Cayman Chemical, USA, Catalog no. 10004947). RAW264.7 cells were cultured in high‐glucose DMEM (MeilunBio, China, Catalog no. MA0212) containing 10% FBS and 1% penicillin–streptomycin. All cells were incubated at 37°C under 5% CO_2_.

For polarization, M1 macrophages: Treated with 100 ng/mL LPS (Sigma‐Aldrich, USA, Catalog no. L2630) and 20 ng/mL IFNγ (Sino Biological, China, Catalog no. 11725‐HANS); M2 macrophages: Treated with 20 ng/mL IL‐4 (Sino Biological, China, Catalog no. 11846‐HNAE) and 20 ng/mL IL‐10 (Sino Biological, China, Catalog no. 10947‐HNAE).

### 4.4 Reagents and Antibodies

PHrodo Red *E. coli* BioParticles (Catalog no. P35361) was purchased from Thermo Fisher Scientific, USA. Latex beads (Catalog no. L3030) were purchased from Sigma‐Aldrich, Canada. Human Type I Collagen Solution (Catalog no. 1200‐01S) was purchased from SouthernBiotech, USA. Cytochalasin D (Catalog no. C10518, an F‐actin polymerization inhibitor) was purchased from PSAITONG, China. MitoTEMPO (Catalog no. GC44206‐1, a mitochondria‐targeted free‐radical scavenger) was purchased from GLPBIO, USA. Clodronate Liposomes (Catalog no. K2721, for macrophage depletion) were purchased from APExBIO, USA. Rotenone (Catalog no. HY‐B1756, a mitochondrial complex I inhibitor) and Diethyl succinate (Catalog no. HY‐Y0836) were purchased from MedChemExpress, USA. Antimycin A (Catalog no. KS1019, a mitochondrial complex III inhibitor) was purchased from Pytbio, China. The antibodies list is provided in Table S1.

### 4.5 Drugs Treatments

For exogenous collagen experiments: M1‐polarized human MDMs and THP‐1 cells were treated with 2.5 mM Human Type I Collagen Solution for 24 h, followed by phagocytosis assay.

For Cytochalasin D experiments: Young human MDMs were treated with 2 μM cytochalasin D for 30 min to disrupt F‐actin networks, followed by phagocytosis assay.

For Clodronate liposomes experiments: Mice received intraperitoneal (i.p.) injections of clodronate liposomes or PBS liposomes (200 μL/mouse) 24 h prior to adoptive transfer of traceable RAW264.7 macrophages transfected with COL1A1‐overexpressing or control plasmids. After an additional 24 h, GFP‐labeled *E. coli* was administered i.p. injections, and macrophage phagocytic activity was quantified by flow cytometry 4 h post‐infection.

For MitoTEMPO experiments: In vitro, human MDMs and THP‐1 cells were pretreated with MitoTEMPO (1–10 μM) for 2 h before functional analysis. For in vivo studies, aged male C57BL/6J mice (*n* = 6/group) were randomly assigned to receive either MitoTEMPO (5 mg/kg, i.p.) or sterile water (control). After 24 h, all mice were challenged with GFP‐ *E. coli* via i.p. injection, and macrophage phagocytic function was quantified by flow cytometry 4 h post‐infection.

### 4.6 Plasmid Constructs and Electroporation

For COL1A1 knockdown, a human‐specific shRNA (target sequence: target sequence sh: Forward‐5′CCGGCAAAGGAGACACTGGTGCTAACTCGAGTTAGCACCTGTGTCTCCTTTGTTTTTG3′, Reverse‐5′AATTCAAAAACAAAGGAGACACTGGTGCTAACTCGAGTTAGCACCTGTGTCTCCTTTG3′) was synthesized (Zhejiang Sunya Biotechnology, China). For overexpression studies, human COL1A1 (NM_000088) and mouse Col1a1 (NM_007742) cDNAs were cloned into pcDNA3.1–3 × Flag vectors, while human FN1 (NM_212482) and COL1A2 (NM_000089) cDNAs were cloned into pcDNA3.1–3 × HA vectors. All constructs were generated by GuanNan Co Ltd. (China). After overnight incubation (37°C, 5% CO_2_), cells were resuspended in Hyclone Electroporation Buffer (MaxCyte, USA) at 20 × 10^5^ cells/mL. Add gene‐specific shRNA or overexpression plasmid and the negative control plasmid into separate aliquots of MaxCyte HyClone buffer to be the total 100 μL. Electroporation was performed using a MaxCyte ATx electroporation instrument (MaxCyte, Gaithersburg, MD, USA). Knockdown and overexpression efficiency were validated by qRT‐PCR and Western blot.

### 4.7 Phagocytosis Assay

For in vitro phagocytosis assay, human M1 macrophages were cultured in 24‐well plates and incubated with pHrodo Red *E. coli* BioParticles (20 μg/mL in PBS) or fluorescent latex beads (2 μL/well) for 2 h. Cells were washed twice with PBS, detached by pipetting, and analyzed via flow cytometry (CytoFLEX, Beckman Coulter, USA). Phagocytic activity (% positive cells) was quantified using FlowJo software (Tree Star, Ashland, OR, USA, version 10.8.1). For in vivo phagocytosis assay, pHrodo *E. coli* BioParticles (100 μg/mL in PBS) were intraperitoneally injected into young and old C57BL/6 mice. After 4 h, peritoneal cells were collected with ice‐cold PBS and stained with anti‐F4/80 (Catalog no. E‐AB‐F0995M, Elabscience, China) and anti‐CD11b (Catalog no. E‐AB‐F1081F, Elabscience, China) antibodies for macrophage identification and phagocytosis analysis by flow cytometry.

### 4.8 RNA Isolation and Quantitative Real‐Time RT‐PCR (qRT‐PCR) Analysis

Total RNA was extracted from macrophages using RNA‐Quick Purification Kit (YiShan Biotech, China) according to the manufacturer's protocol. Reverse transcription (RT) was performed with Fast All‐in‐One RT Kit (Catalog no. ES‐RT001, YiShan Biotech, China). RT‐PCR was performed with a ChamQ SYBR qPCR Master Mix Kit (Catalog no. ES‐RN001, Vazyme, China) on the Bio‐Rad CFX96 real‐time PCR system (Bio‐Rad, USA), and gene expression was normalized to 18 s (human) and β‐actin (mouse) transcripts. Primers are listed in Table S2.

### 4.9 Transcriptome Sequencing

Total RNA from the cells was collected using RNA‐Quick Purification Kit (YiShan Biotech, China) and subjected to genome‐wide transcriptomic analysis using LC‐Bio (Hangzhou, China). Differentially expressed genes (DEGs) were selected with fold change > 2 or fold change < 0.5 and *p*‐value < 0.05. DEGs were then subjected to enrichment analysis of GO functions and KEGG pathways. All bioinformatics were analyzed using the OmicStudio tools (https://www.omicstudio.cn/tool). RNAseq data are available on Gene Expression Omnibus under accession number GSE306336.

### 4.10 Western Blot

Cellular proteins were extracted with radioimmunoprecipitation buffer (RIPA) containing protease and phosphatase inhibitors (Catalog no. GRF102, Epizyme, China). Expression levels were examined following standard Western blotting protocols. Primary antibodies used: anti‐COL1A1 (Catalog no. ab138492, Abcam, USA). Anti‐β‐actin (Catalog no. AC038, ABclonal, China) served as internal controls. Western blot band intensities were quantified using ImageJ software (NIH, USA).

### 4.11 Co‐Immunoprecipitation (Co‐IP) Assay

For co‐immunoprecipitation, cells were lysed with IP Lysis Buffer (Catalog no. RM00022, ABclonal, China) containing protease and phosphatase inhibitors. Lysates were first incubated with Protein A/G magnetic beads (Catalog no. L‐2204, Biolinkedin, China) for 2 h, followed by overnight immunoprecipitation at 4°C with 2 μg anti‐Actin antibody (Catalog no. 66009‐1‐Ig, Proteintech, China) on a rotator. Normal rat IgG (Catalog no. AC011, ABclonal, China) was used as IgG control. The immunocomplexes were washed with IP lysis buffer 5 times, then eluted with loading buffer. Immunoblotting for COL1A1 (Catalog no. ab138492, Abcam, USA) and Annexin A2 (Catalog no. A5311, Selleck Chemicals, USA) was performed following standard procedures for Western blot.

### 4.12 LC–MS/MS Analysis

Two distinct monoclonal antibodies targeting human COL1A1 (Catalog no. 67288‐1‐Ig, Proteintech, China and Catalog no. ab138492, Abcam, USA) were added to the lysate of aged human MDMs for Co‐IP. LC–MS/MS analysis was performed by the core facility, Central Laboratory, the First Affiliated Hospital, Zhejiang University School of Medicine with the mass spectrometry.

### 4.13 Immunofluorescence Microscopy

To visualize intracellular proteins, cells were collected, fixed with 4% paraformaldehyde (PFA) and processed for IF staining. The following primary antibodies were used: anti‐COL1A1 mouse monoclonal antibody (1:400, Catalog no. 67288‐1‐Ig, Proteintech, China). The following secondary antibodies were used: Alexa Fluor‐594 goat anti‐mouse IgG (1:200, Catalog no. A‐11032, Thermo Fisher Scientific, USA). Nuclei were stained with DAPI. The LSM900 system (Carl Zeiss) with a Plan Apochromat ×63/1.40‐NA oil DICIII objective lens (Carl Zeiss) was used to acquire images. Immunofluorescence images were analyzed for mean fluorescence intensity (MFI) using ImageJ software.

### 4.14 Actin Staining of the Cytoskeleton

The cytoskeleton of macrophages was stained with FITC‐phalloidin (Catalog no. 40735ES75, Yeason Biotechnology, China). After fixing with 4% PFA, washing and blocking with human FcR blocking, cells were sequentially incubated with a FITC‐phalloidin solution (100 nM) at room temperature in the dark for 30 min and DAPI at 37°C. Finally, images were taken under confocal microscopy.

### 4.15 Mitochondria Membrane Potential

Human MDMs were loaded with 500 nM MitoTracker Red (Catalog no. C1049B, Beyotime, China) for 30 min at 37°C to detect mitochondrial membrane potential, followed by a wash with warmed complete culture medium. The fluorescence intensity was then measured by flow cytometry.

### 4.16 Measurement of Mitochondrial ROS

Mitochondrial ROS were detected by MitoSOX Red (Catalog no. S0061S, Beyotime, China). MitoSOX Red was diluted in serum‐free RPMI to a final concentration of 5 μM and incubated with the cells for 25 min at 37°C in the dark before flow cytometry and immunofluorescence microscopy.

### 4.17 Fluorescence Recovery After Photobleaching (FRAP) Analysis of Actin Dynamics in Human Macrophages

M1‐polarized HMDMs from young and aged donors were stained with CellMask Actin Tracking Stain (Catalog no. A57243, Thermo Fisher Scientific, USA). FRAP was performed only on the FITC channel using a Zeiss LSM confocal microscope (40× objective, 512 × 512 pixels). Three ROIs were photobleached to 30% initial intensity using 100% 488 nm laser power, with one non‐bleached (ROI 4) and one background ROI (ROI 5) as controls. Prebleach fluorescent signal of FITC was acquired using a 488 nm line at 2.2% laser power. Post‐bleach images were acquired every 3 s for a total of 70 cycles at 2.2% laser power. Fluorescence intensities were background‐subtracted, normalized against the non‐bleached control.

### 4.18 Polymerization and Depolymerization Assays

For polymerization assays, actin (2 μM, 5% pyrenyl labeled, Catalog no. AP05‐A, Cytoskeleton, USA) was polymerized in 10 mM Tris–HCl at pH 8.0, 50 mM KCl, 1 mM MgCl_2_, 0.2 mM ATP in the presence of 10 μM CaCl_2_, 1 mM EGTA, 10 mM DTT (pH 7.0) (F‐buffer) and in the presence or absence of COL1A1 protein (Catalog no. RP01886, ABclonal, China) at a final concentration of 400 nM. For depolymerization assays, actin was polymerized in F‐buffer at 4°C overnight, then incubated with COL1A1 protein with an actin final concentration of 400 nM for 2 min. Depolymerization was initiated by diluting F‐actin to 100 nM using F‐buffer. Fluorescence changes were recorded as a function of time on a fluorescence spectrometer (BioTeK Synergy Neo2).

### 4.19 Quantification and Statistical Analysis

Statistical analyses were performed using GraphPad Prism software (GraphPad Software). To compare data within two groups, the paired Wilcoxon test or the Mann–Whitney test was used when the sample size per group was > 5. Parametric *t*‐test was only used if the sample size per group was ≤ 5. To adjust for multiple testing, we used Hochberg's step‐down method to control for a family‐wise‐error rate at the 0.05 level. One‐way ANOVA was used and pair‐wise comparison using Tukey's method was applied for comparisons between 3 or more groups. All data points were included in the analysis and no outliers were detected using Grubbs' test. All data are presented as mean ± SEM. *p < 0.05, **p < 0.01, ***p < 0.001 and statistical parameters are presented in each figure legend.

## Author Contributions

Yuming Wang and Xin Xu are co‐first authors and are involved in all the experiments and data acquisition. Nuanqin Shen, Ping Han, Liuyi Wu, and Yi Jin helped the first authors with experiments. Yiming Sun analyzed the RNAseq data. Administrative, technical, or material support was provided by Lanlan Xiao and Jinyou Li. Lan Wang, Yunmei Yang, Qin Zhang, and Weiqian Chen critically revised the manuscript. Bowen Wu and Chaohui Yu conceived the study, designed experiments, secured funding, and wrote the manuscript.

## Funding

This work is supported by the National Natural Science Foundation of China (No. 82371800, No. 82271588), the Zhejiang Provincial Natural Science Foundation of China (No. LY24HO30003).

## Conflicts of Interest

The authors declare no conflicts of interest.

## Supporting information


**Figure S1:** (A) Violin plots showing the proportion of monocytes among total cells derived from scRNA‐seq analysis across 317 samples, stratified by age group (Synapse ID: syn49637038). (B) Flow cytometry quantification of latex bead phagocytosis by MDMs from young and elderly donors under unopsonized, complement mediated (human serum), and Fc mediated (human IgG) conditions (*n* = 5 each).
**Figure S2:** (A–C) Gene set enrichment analysis (GSEA) plots for selected pathways. Normalized enrichment score (NES) and *p*‐values are indicated. (D) Relative mRNA level of phagocytic receptors in young and older hMDMs measured by RT‐PCR (*n* = 6 each). (E, F) Expression of COL1A1 (E) and COL1A2 (F) in day‐0 circulating monocytes versus M1 macrophages. Bulk RNA‐seq data from GSE243175. (G) Western blot analysis reveals COL1A1 expression in cell lysates but not in culture supernatants of hMDMs obtained from young and older donors, indicating the absence of mature collagen I secretion.
**Figure S3:** PISA analysis of protein–protein interaction (ACTB octamer and COL1A1) were updated for analysis. (A–G) Represent actin monomer, and I represent COL1A1.
**Figure S4:** (A, B) Flow cytometry quantification of fluorescence‐labeled latex beads (A; *n* = 7 each) and pHrodo‐*E. coli* (B; *n* = 6 each) phagocytosis in young MDMs treated with CytoD (2 μM for 30 min). (C) ANXA2‐Actin binding in THP‐1 cells transfected with either NC or pcDNA3.1‐COL1A1 detected by Co‐IP assay. (D) Confocal microscopy of THP‐1 derived macrophage cell transfected with plasmid expressing GFP‐COL1A1 or control plasmid, F‐actin was stained with phalloidin.
**Figure S5:** (A–F) Correlation between COL1A1 gene expression and genes encoding components of the mitochondrial electron transport chain (ETC) and the mitochondrial fusion protein MFN2 gene expression by linear regression. (G–J) Correlation between COL1A1 gene expression and mitochondrial housekeeping genes and the fission protein DRP1 gene expression by linear regression.
**Figure S6:** (A–F) qRT‐PCR analysis of collagen family mRNA expression in THP‐1 cells treated with H_2_O_2_ (10 μM, 24 h; *n* = 3 each). (G, H) Quantification of Col1a1 MFI (G) and F‐actin MFI (H) in small peritoneal macrophages (SPMs) isolated from LPS‐challenged mice treated with mitoTEMPO (5 mg/kg, 24 h) compared to the control group (*n* = 5 each).
**Table S1:** Antibodies used in this study.
**Table S2:** Primers for qPCR.
**Table S3:** Information of study subjects.

## Data Availability

The RNAseq data generated in this study has been deposited at the Gene Expression Omnibus and is publicly accessible (accession: GSE306336). All data used to generate the statistical analyses and figures in this manuscript are available from the corresponding author upon reasonable request.

## References

[acel70594-bib-0001] Allen, L. A. , and A. Aderem . 1996. “Molecular Definition of Distinct Cytoskeletal Structures Involved in Complement‐ and Fc Receptor‐Mediated Phagocytosis in Macrophages.” Journal of Experimental Medicine 184: 627–637. 8760816 10.1084/jem.184.2.627 PMC2192718

[acel70594-bib-0002] Ardavin, C. , N. Alvarez‐Ladron , M. Ferriz , A. Gutierrez‐Gonzalez , and A. Vega‐Perez . 2023. “Mouse Tissue‐Resident Peritoneal Macrophages in Homeostasis, Repair, Infection, and Tumor Metastasis.” Advanced Science 10: e2206617. 36658699 10.1002/advs.202206617 PMC10104642

[acel70594-bib-0003] Canan, C. H. , N. S. Gokhale , B. Carruthers , et al. 2014. “Characterization of Lung Inflammation and Its Impact on Macrophage Function in Aging.” Journal of Leukocyte Biology 96: 473–480. 24935957 10.1189/jlb.4A0214-093RR PMC4632167

[acel70594-bib-0004] Caron, E. , and A. Hall . 1998. “Identification of Two Distinct Mechanisms of Phagocytosis Controlled by Different Rho GTPases.” Science 282: 1717–1721. 9831565 10.1126/science.282.5394.1717

[acel70594-bib-0005] Casey, A. M. , D. G. Ryan , H. A. Prag , et al. 2025. “Pro‐Inflammatory Macrophages Produce Mitochondria‐Derived Superoxide by Reverse Electron Transport at Complex I That Regulates IL‐1beta Release During NLRP3 Inflammasome Activation.” Nature Metabolism 7: 493–507. 10.1038/s42255-025-01224-x PMC11946910 39972217

[acel70594-bib-0006] Cen, X. , Y. Chen , X. Xu , et al. 2020. “Pharmacological Targeting of MCL‐1 Promotes Mitophagy and Improves Disease Pathologies in an Alzheimer's Disease Mouse Model.” Nature Communications 11: 5731. 10.1038/s41467-020-19547-6 PMC7665171 33184293

[acel70594-bib-0007] Chen, Q. , E. J. Vazquez , S. Moghaddas , C. L. Hoppel , and E. J. Lesnefsky . 2003. “Production of Reactive Oxygen Species by Mitochondria: Central Role of Complex III.” Journal of Biological Chemistry 278: 36027–36031. 12840017 10.1074/jbc.M304854200

[acel70594-bib-0008] De Maeyer, R. P. H. , R. C. van de Merwe , R. Louie , et al. 2020. “Blocking Elevated p38 MAPK Restores Efferocytosis and Inflammatory Resolution in the Elderly.” Nature Immunology 21: 615–625. 32251403 10.1038/s41590-020-0646-0 PMC7983074

[acel70594-bib-0009] Diakonova, M. , G. Bokoch , and J. A. Swanson . 2002. “Dynamics of Cytoskeletal Proteins During Fcgamma Receptor‐Mediated Phagocytosis in Macrophages.” Molecular Biology of the Cell 13: 402–411. 11854399 10.1091/mbc.01-05-0273 PMC65636

[acel70594-bib-0010] Fix, D. K. , H. A. Ekiz , J. J. Petrocelli , et al. 2021. “Disrupted Macrophage Metabolic Reprogramming in Aged Soleus Muscle During Early Recovery Following Disuse Atrophy.” Aging Cell 20: e13448. 34365717 10.1111/acel.13448 PMC8441489

[acel70594-bib-0011] Flannagan, R. S. , J. Canton , W. Furuya , M. Glogauer , and S. Grinstein . 2014. “The Phosphatidylserine Receptor TIM4 Utilizes Integrins as Coreceptors to Effect Phagocytosis.” Molecular Biology of the Cell 25: 1511–1522. 24623723 10.1091/mbc.E13-04-0212 PMC4004599

[acel70594-bib-0012] Freeman, S. A. , A. Vega , M. Riedl , et al. 2018. “Transmembrane Pickets Connect Cyto‐ and Pericellular Skeletons Forming Barriers to Receptor Engagement.” Cell 172: 305–317. 29328918 10.1016/j.cell.2017.12.023 PMC5929997

[acel70594-bib-0013] Gavazzi, G. , and K. H. Krause . 2002. “Ageing and Infection.” Lancet Infectious Diseases 2: 659–666. 12409046 10.1016/s1473-3099(02)00437-1

[acel70594-bib-0014] Goode, B. L. , J. Eskin , and S. Shekhar . 2023. “Mechanisms of Actin Disassembly and Turnover.” Journal of Cell Biology 222: e202309021. 37948068 10.1083/jcb.202309021 PMC10638096

[acel70594-bib-0015] Guan, F. , R. Wang , Z. Yi , et al. 2025. “Tissue Macrophages: Origin, Heterogenity, Biological Functions, Diseases and Therapeutic Targets.” Signal Transduction and Targeted Therapy 10: 93. 40055311 10.1038/s41392-025-02124-y PMC11889221

[acel70594-bib-0016] Hachim, D. , N. Wang , S. T. Lopresti , et al. 2017. “Effects of Aging Upon the Host Response to Implants.” Journal of Biomedical Materials Research. Part A 105: 1281–1292. 28130823 10.1002/jbm.a.36013 PMC5963506

[acel70594-bib-0017] Hanna, R. N. , C. Cekic , D. Sag , et al. 2015. “Patrolling Monocytes Control Tumor Metastasis to the Lung.” Science 350: 985–990. 26494174 10.1126/science.aac9407 PMC4869713

[acel70594-bib-0018] Hayes, M. J. , D. Shao , M. Bailly , and S. E. Moss . 2006. “Regulation of Actin Dynamics by Annexin 2.” EMBO Journal 25: 1816–1826. 16601677 10.1038/sj.emboj.7601078 PMC1456940

[acel70594-bib-0019] Hearps, A. C. , G. E. Martin , T. A. Angelovich , et al. 2012. “Aging Is Associated With Chronic Innate Immune Activation and Dysregulation of Monocyte Phenotype and Function.” Aging Cell 11: 867–875. 22708967 10.1111/j.1474-9726.2012.00851.x

[acel70594-bib-0020] Higashimoto, Y. , Y. Fukuchi , Y. Shimada , et al. 1993. “The Effects of Aging on the Function of Alveolar Macrophages in Mice.” Mechanisms of Ageing and Development 69: 207–217. 8412370 10.1016/0047-6374(93)90024-l

[acel70594-bib-0021] Huang, X. , L. Zhao , and R. Peng . 2022. “Hypoxia‐Inducible Factor 1 and Mitochondria: An Intimate Connection.” Biomolecules 13, no. 1: 50. 36671435 10.3390/biom13010050 PMC9855368

[acel70594-bib-0022] Isagawa, T. , M. S. Morioka , H. Semba , et al. 2025. “LPS Induces Limited Activation of Hypoxia‐Inducible Factor‐1alpha in Macrophages.” Journal of Biological Chemistry 301: 110932. 41232669 10.1016/j.jbc.2025.110932 PMC12720325

[acel70594-bib-0023] Kline, K. A. , and D. M. Bowdish . 2016. “Infection in an Aging Population.” Current Opinion in Microbiology 29: 63–67. 26673958 10.1016/j.mib.2015.11.003

[acel70594-bib-0024] Lacy, M. M. , D. Baddeley , and J. Berro . 2019. “Single‐Molecule Turnover Dynamics of Actin and Membrane Coat Proteins in Clathrin‐Mediated Endocytosis.” eLife 8: e52355. 31855180 10.7554/eLife.52355 PMC6977972

[acel70594-bib-0025] Li, N. , K. Ragheb , G. Lawler , et al. 2003. “Mitochondrial Complex I Inhibitor Rotenone Induces Apoptosis Through Enhancing Mitochondrial Reactive Oxygen Species Production.” Journal of Biological Chemistry 278: 8516–8525. 12496265 10.1074/jbc.M210432200

[acel70594-bib-0026] Li, Z. , Y. Jiao , E. K. Fan , et al. 2017. “Aging‐Impaired Filamentous Actin Polymerization Signaling Reduces Alveolar Macrophage Phagocytosis of Bacteria.” Journal of Immunology 199: 3176–3186. 10.4049/jimmunol.1700140 PMC5679440 28947541

[acel70594-bib-0027] Lima, T. , T. Y. Li , A. Mottis , and J. Auwerx . 2022. “Pleiotropic Effects of Mitochondria in Aging.” Nat Aging 2: 199–213. 37118378 10.1038/s43587-022-00191-2

[acel70594-bib-0028] Linehan, E. , Y. Dombrowski , R. Snoddy , P. G. Fallon , A. Kissenpfennig , and D. C. Fitzgerald . 2014. “Aging Impairs Peritoneal but Not Bone Marrow‐Derived Macrophage Phagocytosis.” Aging Cell 13: 699–708. 24813244 10.1111/acel.12223 PMC4326936

[acel70594-bib-0029] Migliaccio, G. , J. Morikka , G. del Giudice , et al. 2024. “Methylation and Transcriptomic Profiling Reveals Short Term and Long Term Regulatory Responses in Polarized Macrophages.” Computational and Structural Biotechnology Journal 25: 143–152. 39257962 10.1016/j.csbj.2024.08.018 PMC11385784

[acel70594-bib-0030] Mole, D. R. , C. Blancher , R. R. Copley , et al. 2009. “Genome‐Wide Association of Hypoxia‐Inducible Factor (HIF)‐1alpha and HIF‐2alpha DNA Binding With Expression Profiling of Hypoxia‐Inducible Transcripts.” Journal of Biological Chemistry 284: 16767–16775. 19386601 10.1074/jbc.M901790200 PMC2719312

[acel70594-bib-0031] Moss, C. E. , S. A. Johnston , J. V. Kimble , et al. 2024. “Aging‐Related Defects in Macrophage Function Are Driven by MYC and USF1 Transcriptional Programs.” Cell Reports 43: 114073. 38578825 10.1016/j.celrep.2024.114073

[acel70594-bib-0032] Mylvaganam, S. , S. A. Freeman , and S. Grinstein . 2021. “The Cytoskeleton in Phagocytosis and Macropinocytosis.” Current Biology 31: R619–R632. 34033794 10.1016/j.cub.2021.01.036

[acel70594-bib-0033] Narayanan, P. , P. Chatterton , A. Ikeda , et al. 2015. “Length Regulation of Mechanosensitive Stereocilia Depends on Very Slow Actin Dynamics and Filament‐Severing Proteins.” Nature Communications 6: 6855. 10.1038/ncomms7855 PMC4523390 25897778

[acel70594-bib-0034] Natrajan, M. S. , A. G. de la Fuente , A. H. Crawford , et al. 2015. “Retinoid X Receptor Activation Reverses Age‐Related Deficiencies in Myelin Debris Phagocytosis and Remyelination.” Brain 138: 3581–3597. 26463675 10.1093/brain/awv289 PMC4668920

[acel70594-bib-0035] Nimmerjahn, A. , F. Kirchhoff , and F. Helmchen . 2005. “Resting Microglial Cells Are Highly Dynamic Surveillants of Brain Parenchyma In Vivo.” Science 308: 1314–1318. 15831717 10.1126/science.1110647

[acel70594-bib-0036] Onursal, C. , E. Dick , I. Angelidis , H. B. Schiller , and C. A. Staab‐Weijnitz . 2021. “Collagen Biosynthesis, Processing, and Maturation in Lung Ageing.” Frontiers in Medicine (Lausanne) 8: 593874. 10.3389/fmed.2021.593874 PMC8172798 34095157

[acel70594-bib-0037] Palmer, A. K. , and M. D. Jensen . 2022. “Metabolic Changes in Aging Humans: Current Evidence and Therapeutic Strategies.” Journal of Clinical Investigation 132: e158451. 35968789 10.1172/JCI158451 PMC9374375

[acel70594-bib-0038] Peradinovic, J. , N. Mohovic , K. Bulic , A. Markovinovic , R. Cimbro , and I. Munitic . 2023. “Ageing‐Induced Decline in Primary Myeloid Cell Phagocytosis Is Unaffected by Optineurin Insufficiency.” Biology‐Basel 12, no. 2: 240. 36829517 10.3390/biology12020240 PMC9953198

[acel70594-bib-0039] Rawji, K. S. , J. Kappen , W. Tang , et al. 2018. “Deficient Surveillance and Phagocytic Activity of Myeloid Cells Within Demyelinated Lesions in Aging Mice Visualized by Ex Vivo Live Multiphoton Imaging.” Journal of Neuroscience 38: 1973–1988. 29363580 10.1523/JNEUROSCI.2341-17.2018 PMC6705888

[acel70594-bib-0040] Rawji, K. S. , A. M. H. Young , T. Ghosh , et al. 2020. “Niacin‐Mediated Rejuvenation of Macrophage/Microglia Enhances Remyelination of the Aging Central Nervous System.” Acta Neuropathologica 139: 893–909. 32030468 10.1007/s00401-020-02129-7 PMC7181452

[acel70594-bib-0041] Rougerie, P. , V. Miskolci , and D. Cox . 2013. “Generation of Membrane Structures During Phagocytosis and Chemotaxis of Macrophages: Role and Regulation of the Actin Cytoskeleton.” Immunological Reviews 256: 222–239. 24117824 10.1111/imr.12118 PMC3806206

[acel70594-bib-0042] Rzadzinska, A. K. , M. E. Schneider , C. Davies , G. P. Riordan , and B. Kachar . 2004. “An Actin Molecular Treadmill and Myosins Maintain Stereocilia Functional Architecture and Self‐Renewal.” Journal of Cell Biology 164: 887–897. 15024034 10.1083/jcb.200310055 PMC2172292

[acel70594-bib-0043] Schnoor, M. , P. Cullen , J. Lorkowski , et al. 2008. “Production of Type VI Collagen by Human Macrophages: A New Dimension in Macrophage Functional Heterogeneity.” Journal of Immunology 180: 5707–5719. 10.4049/jimmunol.180.8.5707 18390756

[acel70594-bib-0044] Simoes, F. C. , T. J. Cahill , A. Kenyon , et al. 2020. “Macrophages Directly Contribute Collagen to Scar Formation During Zebrafish Heart Regeneration and Mouse Heart Repair.” Nature Communications 11: 600. 10.1038/s41467-019-14263-2 PMC6992796 32001677

[acel70594-bib-0045] Tabata Fukushima, C. , I. S. Dancil , H. Clary , N. Shah , S. M. Nadtochiy , and P. S. Brookes . 2024. “Reactive Oxygen Species Generation by Reverse Electron Transfer at Mitochondrial Complex I Under Simulated Early Reperfusion Conditions.” Redox Biology 70: 103047. 38295577 10.1016/j.redox.2024.103047 PMC10844975

[acel70594-bib-0046] Tang, B. L. 2001. “ADAMTS: A Novel Family of Extracellular Matrix Proteases.” International Journal of Biochemistry & Cell Biology 33: 33–44. 11167130 10.1016/s1357-2725(00)00061-3

[acel70594-bib-0047] Terekhova, M. , A. Swain , P. Bohacova , et al. 2023. “Single‐Cell Atlas of Healthy Human Blood Unveils Age‐Related Loss of NKG2C(+)GZMB(−)CD8(+) Memory T Cells and Accumulation of Type 2 Memory T Cells.” Immunity 56: 2836–2854. 37963457 10.1016/j.immuni.2023.10.013

[acel70594-bib-0048] Tsitoura, E. , A. Trachalaki , E. Vasarmidi , et al. 2021. “Collagen 1a1 Expression by Airway Macrophages Increases in Fibrotic ILDs and Is Associated With FVC Decline and Increased Mortality.” Frontiers in Immunology 12: 645548. 34867934 10.3389/fimmu.2021.645548 PMC8635798

[acel70594-bib-0049] Watanabe, N. , and T. J. Mitchison . 2002. “Single‐Molecule Speckle Analysis of Actin Filament Turnover in Lamellipodia.” Science 295: 1083–1086. 11834838 10.1126/science.1067470

[acel70594-bib-0050] Watanabe, S. , M. Alexander , A. V. Misharin , and G. R. S. Budinger . 2019. “The Role of Macrophages in the Resolution of Inflammation.” Journal of Clinical Investigation 129: 2619–2628. 31107246 10.1172/JCI124615 PMC6597225

[acel70594-bib-0051] Wong, C. K. , C. A. Smith , K. Sakamoto , N. Kaminski , J. L. Koff , and D. R. Goldstein . 2017. “Aging Impairs Alveolar Macrophage Phagocytosis and Increases Influenza‐Induced Mortality in Mice.” Journal of Immunology 199: 1060–1068. 10.4049/jimmunol.1700397 PMC5557035 28646038

