# Supplementary material for: Aging Impairs Macrophage Phagocytosis Through Mitochondrial ROS‐Induced Collagen Production
Source: Aging Cell. 2026 Jun 15;25(6):e70594. doi: 10.1111/acel.70594 (PMC13269868; doi:10.1111/acel.70594)
Supplement: Supplementary file 1 — Figure S1: (A) Violin plots showing the proportion of monocytes among total cells derived from scRNA‐seq analysis across 317 samples, stratified by age group (Synapse ID: syn49637038). (B) Flow cytometry quantification of latex bead phagocytosis by MDMs from young and elderly donors under unopsonized, complement mediated (human serum), and Fc mediated (human IgG) conditions (n = 5 each). Figure S2: (A–C) Gene set enrichment analysis (GSEA) plots for selected pathways. Normalized enrichment score (NES) and p‐values are indicated. (D) Relative mRNA level of phagocytic receptors in young and older hMDMs measured by RT‐PCR (n = 6 each). (E, F) Expression of COL1A1 (E) and COL1A2 (F) in day‐0 circulating monocytes versus M1 macrophages. Bulk RNA‐seq data from GSE243175. (G) Western blot analysis reveals COL1A1 expression in cell lysates but not in culture supernatants of hMDMs obtained from young and older donors, indicating the absence of mature collagen I secretion. Figure S3: PISA analysis of protein–protein interaction (ACTB octamer and COL1A1) were updated for analysis. (A–G) Represent actin monomer, and I represent COL1A1. Figure S4: (A, B) Flow cytometry quantification of fluorescence‐labeled latex beads (A; n = 7 each) and pHrodo‐E. coli (B; n = 6 each) phagocytosis in young MDMs treated with CytoD (2 μM for 30 min). (C) ANXA2‐Actin binding in THP‐1 cells transfected with either NC or pcDNA3.1‐COL1A1 detected by Co‐IP assay. (D) Confocal microscopy of THP‐1 derived macrophage cell transfected with plasmid expressing GFP‐COL1A1 or control plasmid, F‐actin was stained with phalloidin. Figure S5: (A–F) Correlation between COL1A1 gene expression and genes encoding components of the mitochondrial electron transport chain (ETC) and the mitochondrial fusion protein MFN2 gene expression by linear regression. (G–J) Correlation between COL1A1 gene expression and mitochondrial housekeeping genes and the fission protein DRP1 gene expression by linear regression. Figure S6: ( [file ACEL-25-e70594-s001.docx]

**Supplemental Figures**


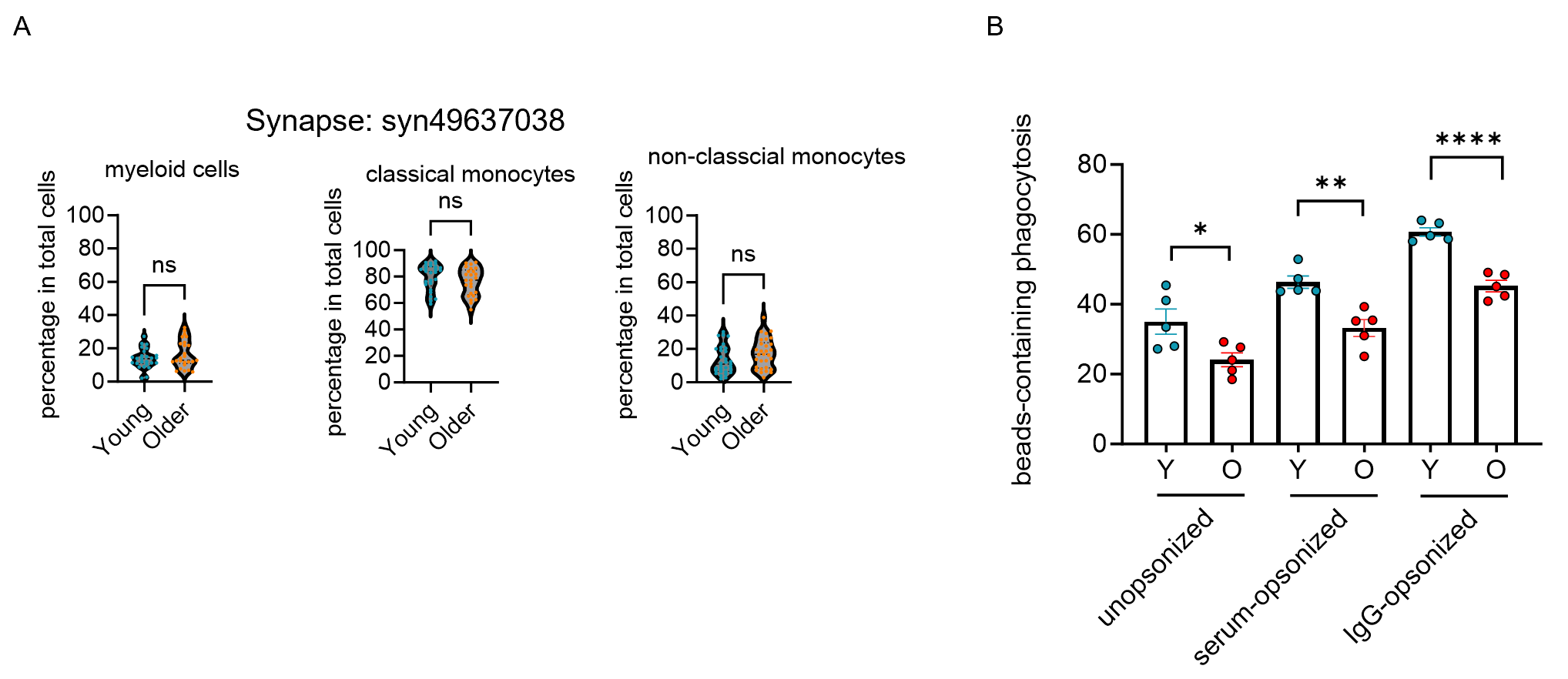


**Fig. S1** (A) Violin plots showing the proportion of monocytes among total cells derived from scRNA-seq analysis across 317 samples, stratified by age group. (Synapse ID: syn49637038). (B) Flow cytometry quantification of latex bead phagocytosis by MDMs from young and elderly donors under unopsonized, complement mediated (human serum), and Fc mediated (human IgG) conditions. (n=5 each)


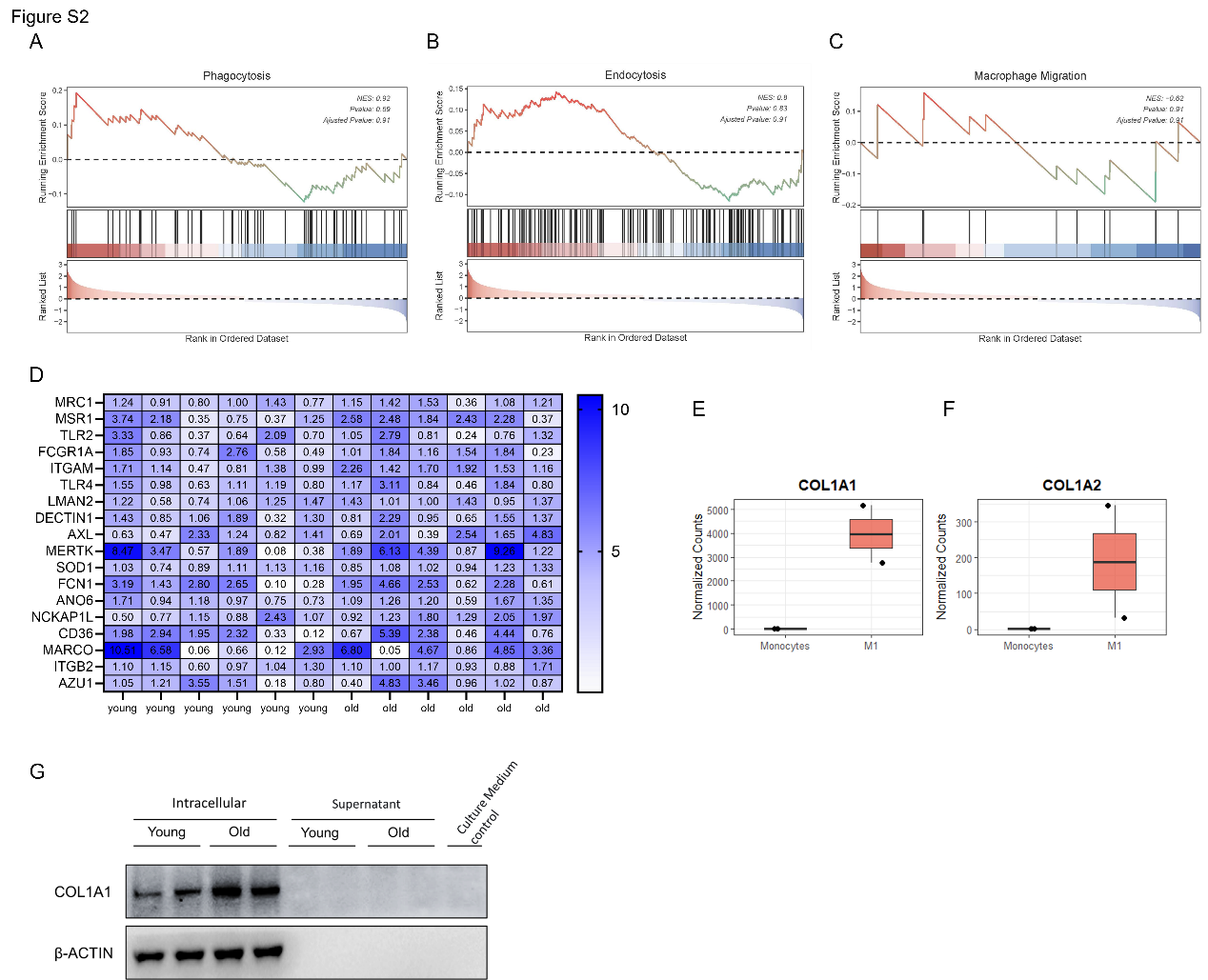


**Fig. S2** (A-C) Gene set enrichment analysis (GSEA) plots for selected pathways. Normalized enrichment score (NES) and P-values are indicated. (D) Relative mRNA level of phagocytic receptors in young and older hMDMs measured by RT-PCR (n =6 each). (E-F) Expression of COL1A1 (E) and COL1A2 (F) in day‑0 circulating monocytes versus M1 macrophages. Bulk RNA‑seq data from GSE243175. (G) Western blot analysis reveals COL1A1 expression in cell lysates but not in culture supernatants of hMDMs obtained from young and older donors, indicating the absence of mature collagen I secretion.


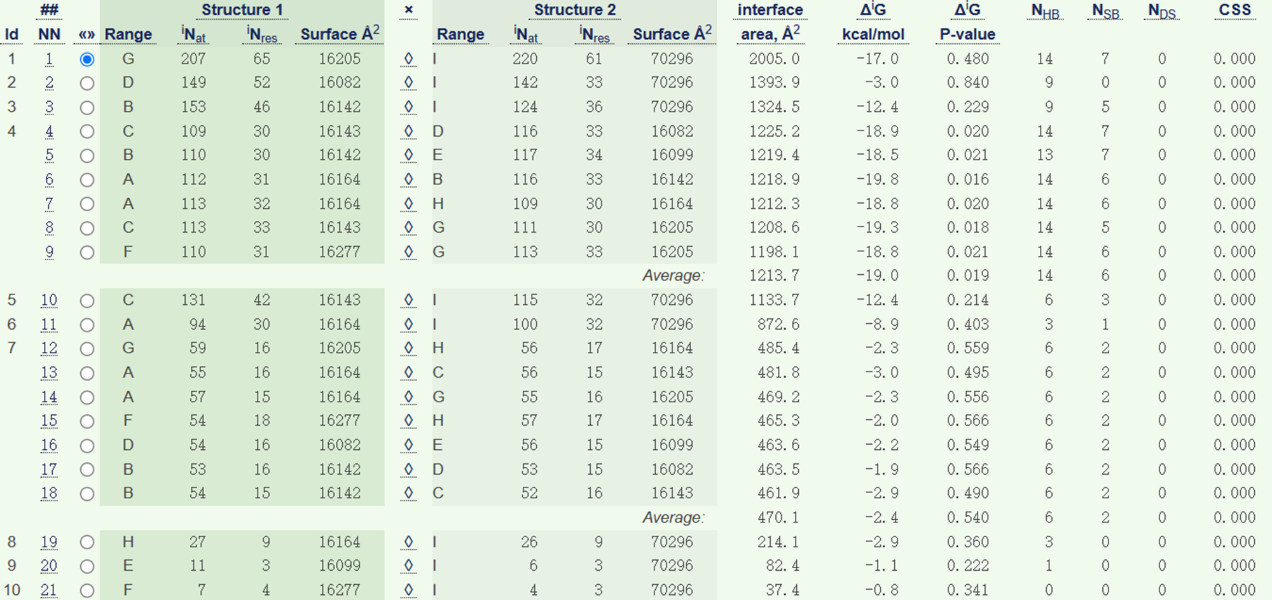


**Fig. S3** PISA analysis of protein-protein interaction (ACTB octamer and COL1A1 were updated for analysis. A-G represent actin monomer, and I represent COL1A1.


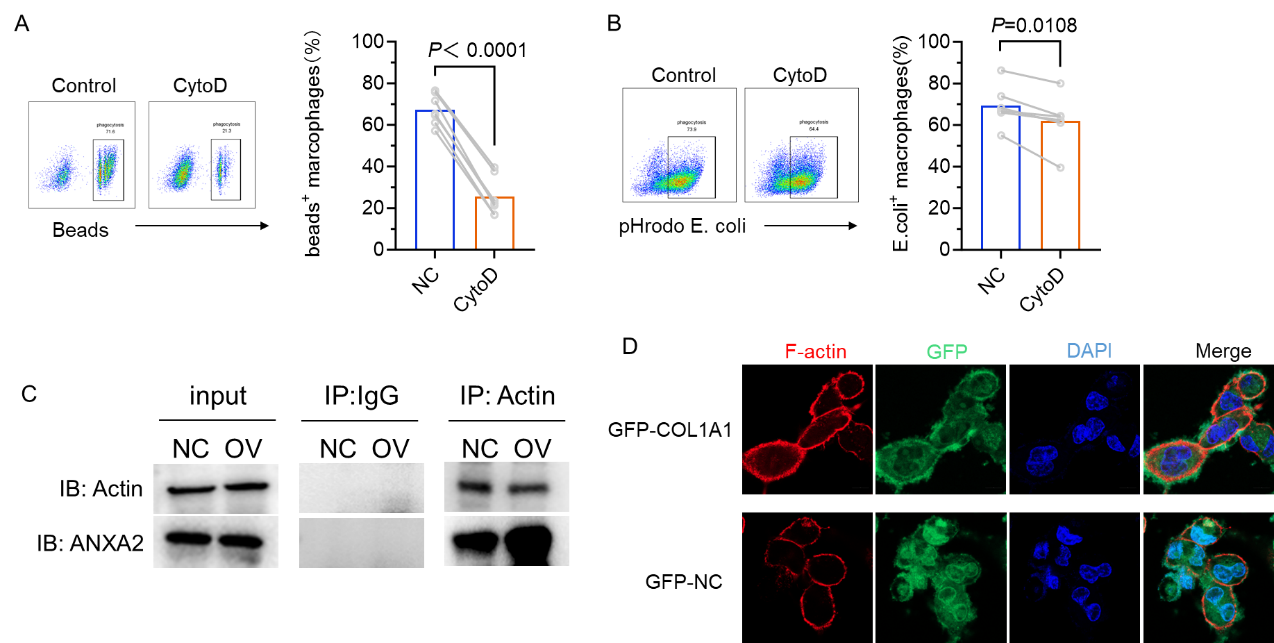


**Fig. S4** (A-B) Flow cytometry quantification of fluorescence-labeled latex beads (A; n=7 each) and pHrodo-E.coli (B; n=6 each) phagocytosis in young MDMs treated with CytoD (2μM for 30min). (C) ANXA2-Actin binding in THP-1 cells transfected with either NC or pcDNA3.1-COL1A1 detected by Co-IP assay. (D) Confocal microscopy of THP-1 derived macrophage cell transfected with plasmid expressing GFP-COL1A1 or control plasmid, F-actin was stained with phalloidin.


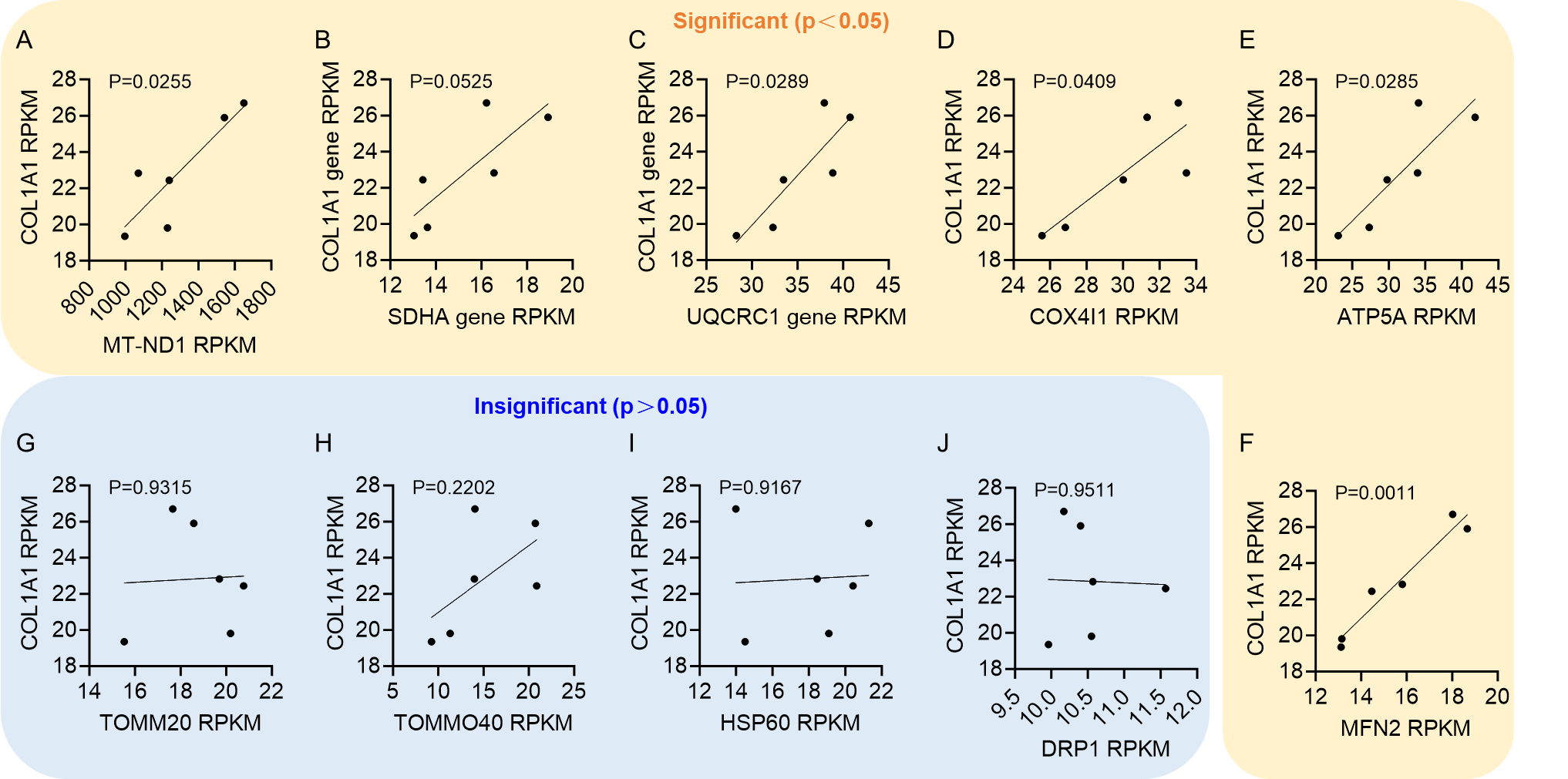


**Fig. S5** (A-F) Correlation between COL1A1 gene expression and genes encoding components of the mitochondrial electron transport chain (ETC) and the mitochondrial fusion protein MFN2 gene expression by linear regression. (G-J) Correlation between COL1A1 gene expression and mitochondrial housekeeping genes and the fission protein DRP1 gene expression by linear regression.

**
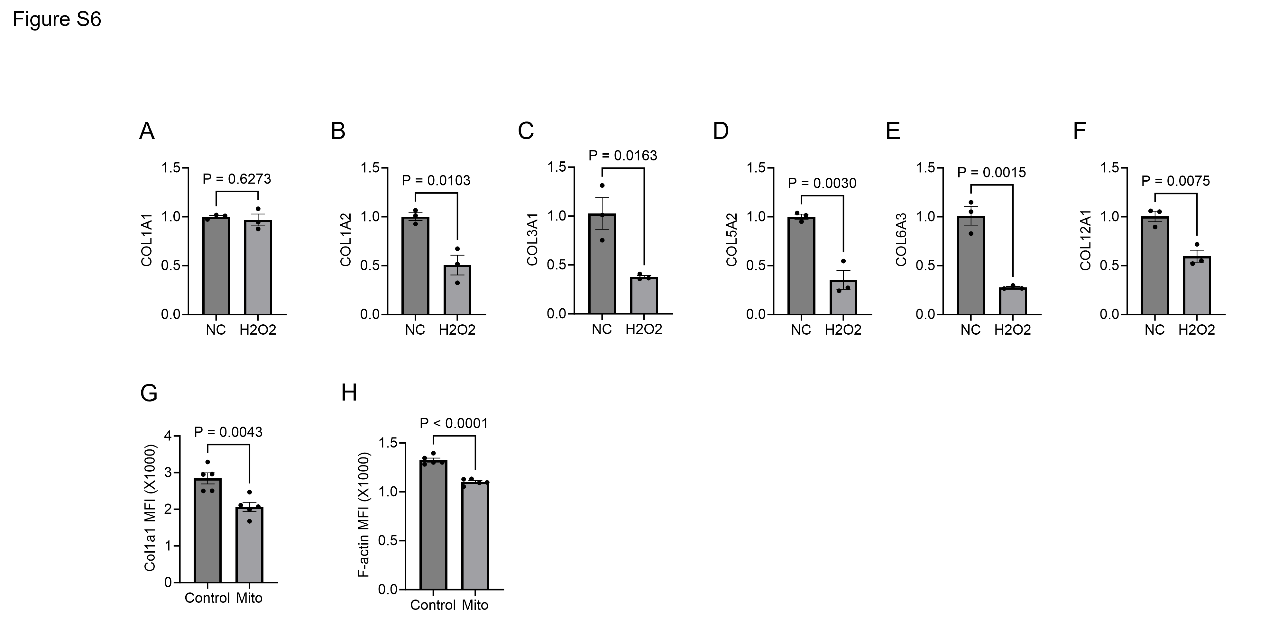
**

**Fig. S6** （A-F）qRT-PCR analysis of collagen family mRNA expression in THP-1 cells treated with H_2_O_2_ (10 μM, 24h; n=3 each). (G-H) Quantification of Col1a1 MFI (G) and F‑actin MFI (H) in small peritoneal macrophages (SPMs) isolated from LPS‑challenged mice treated with mitoTEMPO (5 mg/kg, 24 h) compared to the control group. (n=5 each)

**Supplementary table 1 - Antibodies used in this study**

**Supplementary table 2 – Primers for qPCR**

| **Species** | **Primer** | **Forward** | **Reverse** |
| --- | --- | --- | --- |
| mouse | COL1A1 | CGATGGATTCCCGTTCGAGT | CGATCTCGTTGGATCCCTGG |
| mouse | ACTB | CACTGTCGAGTCGCGTCC | TCATCCATGGCGAACTGGTG |
| human | COL1A1 | CAGGTCTCGGTCATGGTACCT | GTCGAGGGCCAAGACGAA |
| human | 18s | GCAGAATCCACGCCAGTACAAG | GCTTGTTGTCCAGACCATTGGC |
| human | MRC1 | GTGATGGGACCCCTGTAACG | CTGCCCAGTACCCATCCTTG |
| human | MSR1 | CTCACTTTGGACAAGGTACTGG | GAGCAGCGATTTCATAGTTGTG |
| human | TLR2 | CGTTCTCTCAGGTGACTGCTC | CCCTGTCTTCCTGCCTTCAC |
| human | FCGR1A | AAGAACAGCTGCAGGAAGGG | GTTTGTACGCAGTGCTCACG |
| human | ITGAM | TTGTGAGGTCCCAGACGGA | TCTGGGCAGAGTCCTGCT |
| human | TLR4 | ATGCCAGGATGATGTCTGCC | TTTAGGGCCAAGTCTCCACG |
| human | LMAN2 | GGCGACCTGTCTGACAATCA | CAGTCGATGCTCTCCTCGTC |
| human | DECTIN1 | GGGGCTCTCAAGAACAATGGA | GCAGCACACGATCCTTTCTC |
| human | AXL | CCGTGGACCTACTCTGGCT | CCTTGGCGTTATGGGCTTC |
| human | MERTK | CGCTCTGGCGTAGAGCTATC | AGGCTGGGTTGGTGAAAACA |
| human | SOD1 | CGCACACTGGTGGTCCAT | TGGGCGATCCCAATTACACC |
| human | FCN1 | GGACACATGTCCAGAGGTGAA | CATCCCCTTCTCTCCTCGGT |
| human | ANO6 | CGACGATGGGGATATCGTGTT | CCGGGGTTCGAAAATCATGC |
| human | NCKAP1L | GGGGCCAAGCAGGCATTAAA | CTGGTCCTAAATGTTGCGTGC |
| human | CD36 | ACTGAGGACTGCAGTGTAGG | GGTTTCTACAAGCTCTGGTTCTTA |
| human | MARCO | TTCTCCCTAGCTGTGGTGGT | CAGGTGTTCTCCAGGGTGTG |
| human | ITGB2 | TCCCAGTGGAACAATGATAATCC | ACGGCCTTGTCTTCACCAAG |
| human | AZU1 | CTGCTTCCAAAGCCAGAACC | TGAAGCAGCATCAGGTCGTT |

**Supplementary table 3 – Information of study subjects**
